# The recruitment of CD8^+^ T cells through YBX1 stabilization abrogates tumor intrinsic oncogenic role of MIR155HG in lung adenocarcinoma

**DOI:** 10.1038/s41420-024-02102-3

**Published:** 2024-07-23

**Authors:** Rutao Li, Yijian Zhang, Anpeng Wang, Yipeng Feng, Te Zhang, Hui Wang, Yuzhong Chen, Xinnian Yu, Xuming Song, HanLin Ding, Lin Xu, Gaochao Dong, Feng Jiang

**Affiliations:** 1 Department of Thoracic Surgery, Nanjing Medical University Affiliated Cancer Hospital & Jiangsu Cancer Hospital & Jiangsu Institute of Cancer Research, Nanjing, China; 2 Jiangsu Key Laboratory of Molecular and Translational Cancer Research, Cancer Institute of Jiangsu Province, Nanjing, China; 3 The Fourth Clinical College of Nanjing Medical University, Nanjing, China; 4 Department of Geriatric Oncology, The First Affiliated Hospital of Nanjing Medical University, Nanjing, China; 5 Department of Oncology, Nanjing Medical University Affiliated Cancer Hospital & Jiangsu Cancer Hospital & Jiangsu Institute of Cancer Research, Nanjing, China; 6 Collaborative Innovation Center for Cancer Personalized Medicine, Nanjing Medical University, Nanjing, China

**Keywords:** Cancer microenvironment, Predictive markers

## Abstract

Previous studies revealed that MIR155HG possessed an oncogenic role in many types of tumors including lung adenocarcinoma (LUAD), along with higher expression in tumors. However, in our study, we observed a positive correlation between MIR155HG expression and overall survival across different cohorts. The transferred PBMC on the NCG mouse model abrogated the tumor intrinsic oncogenic role of MIR155HG in LUAD. Upregulation of MIR155HG positively correlated with CD8^+^ T cell infiltration both in vitro and in vivo, as well as LUAD tissues. Mechanistically, we revealed that MIR155HG increased the cytokine CCL5 expression at the transcriptional level, which depended on the interaction between MIR155HG and YBX1 protein, a novel transcription factor of CCL5, resulting in the more protein stability of YBX1 through dampening ubiquitination. Additionally, we also observed that MIR155 could increase PD-L1 expression to hamper the activity of recruited CD8^+^ T cells, which could be rescued through PD-L1 mAb addition. Finally, we uncovered that patients with high MIR155HG expression had a higher response rate to immunotherapy, and the combination of MIR155HG overexpression and PD-L1 mAb increased the efficacy of PD-L1 mAb. Together, our study provides a novel biomarker and potential combination treatment strategy for patients who received immunotherapy.

## Introduction

In recent years, immunotherapy, particularly, PD-1/PD-L1 immune checkpoint inhibitors providing a means of utilizing a patient’s own immune system to exterminate cancer cells, has demonstrated substantial benefits for many patients with advanced cancer types. Specifically, PD-1 inhibitor immunotherapy has notably improved a 5-year survival rate in advanced Non-Small Cell Lung Cancer (NSCLC) instances from a dismal rate of less than 5% to approximately 16% [1]. However, the response rate to PD-1 or PD-L1 mAb therapy remains below 40% in most cases [2]. PD-L1 TPS has been widely used to predict the efficacy of immunotherapy in patients with lung adenocarcinoma, but its predictive value for immunotherapy is still insufficient [3]. Therefore, it is urgently needed to identify biomarkers that can predict the efficacy of immunotherapy or to explore alternative treatments such as combination therapy [4].

With the advancement of immunotherapy research, the critical role of the immune microenvironment in immunotherapy has become increasingly recognized [5, 6]. The nature of interactions between tumor cells and immune cells in the tumor microenvironment (TME) dictates the antitumor response [7, 8]. Moreover, immune infiltration in the TME has been established as a crucial factor in tumor development and bears significant implications for the clinical outcomes of cancer patients [9, 10]. As a result, immunotherapies mediated by the regulatory mechanisms of the tumor immune microenvironment offer substantial development prospects. Therefore, understanding the mechanism of regulating immune infiltration within the TME is essential to enhance the effectiveness of immunotherapy and devise new strategies for cancer immunotherapy.

The MIR155 host gene (MIR155HG), a novel member of the lncRNA family, is classified as the primary-miRNA (primiRNA) of miR-155. It has been suggested to have an oncogenic role in various cancers [11–13]. Meanwhile, MIR155HG reportedly encodes a 17-amino acid micropeptide that contributes to DC-driven autoinflammation [14]. With the development of research, Peng and colleagues reports that high expression of MIR155HG was closely related with immune checkpoint molecules expression [15]. For all this, the specific mechanism of MIR155HG in the immune microenvironment of lung adenocarcinoma and its relationship with immunotherapy remain to be clarified.

Here, we observed that MIR155HG is associated with a favorable prognosis in LUAD patients and the existence of immune system abrogates tumor intrinsic oncogenic role of MIR155HG in lung adenocarcinoma by using PBMC-transferred NCG mice. Furtherly, we revealed that the interaction between MIR155HG and YBX1 resulted in upregulation of CCL5 expression to recruit more CD8^+^ T cells in tumor environment. Besides, we investigated the potential of MIR155HG as a biomarker of immunotherapy efficacy prediction in LUAD patients and explored its combination with PD-L1 mAb treatment in mouse models to enhance immunotherapy efficacy, which provides a new strategy and therapeutic opportunity for LUAD patients received immunotherapy.

## Results

### Existence of immune environment abrogates tumor intrinsic oncogenic role of MIR155HG in lung adenocarcinoma

**Fig. 1:**
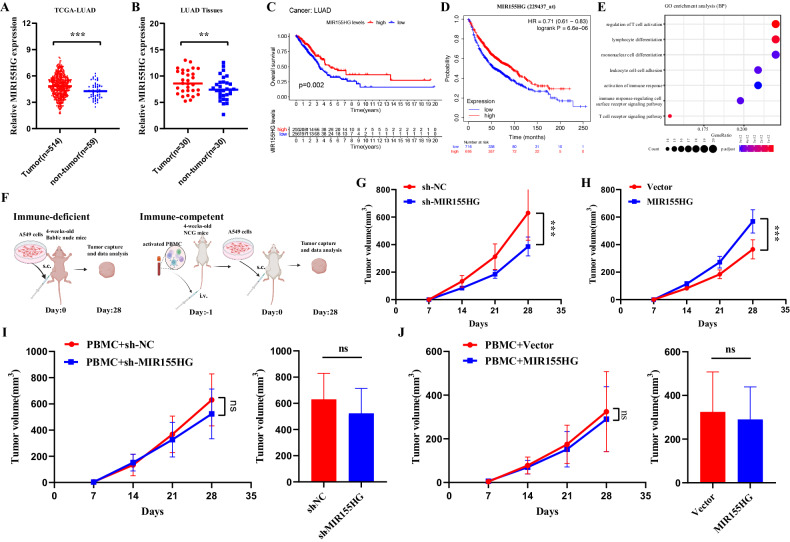
Existence of immune environment abrogates tumor intrinsic oncogenic role of MIR155HG in lung adenocarcinoma. **A** Relative expression levels of MIR155HG in LUAD tissues (*n* = 514) and normal tissues (*n* = 59) from TCGA database. **B** Relative expression levels of MIR155HG in tumor tissues (*n* = 30 tumors) and normal tissues (*n* = 30 samples) from LUAD patients. **C** Kaplan–Meier survival curves of OS from TCGA database according to MIR155HG levels in LUAD tissues. **D** Prognostic values of MIR155HG in LUAD using probes of 229437_s_at. **E** Enriched GO pathway analysis on MIR155HG-related gene signature. **F** Schematic view of mice model in BALB/c nude mice and PBMC-transferred NCG mice respectively. **G** Tumor growth curves of sh-NC and sh-MIR155HG-transfected A549 cells in BALB/c nude mice. **H** Tumor growth curves of Vector and MIR155HG-transfected A549 cells in BALB/c nude mice. **I** Tumor growth curves and tumor volumes of sh-NC and sh-MIR155HG-transfected A549 cells in PBMC-transferred NCG mice. **J** Tumor growth curves and tumor volumes of Vector and MIR155HG-transfected A549 cells in PBMC-transferred NCG mice. Results are presented as mean ± SEM, *n* = 5. ns, not significant; **p < 0.01, ***p < 0.001.

Previous studies have reported that MIR155HG promoted carcinogenesis and disease progression of various solid tumors [11–13]. We initially investigated TCGA datasets of lung adenocarcinoma (LUAD) and GEO dataset (GSE40791), which revealed that MIR155HG expression in LUAD was markedly higher than that in adjacent normal tissues (Figs. 1A and S1A), which was confirmed in a small LUAD cohort with 30 paired samples (Fig. 1B). We also explored the relation between MIR155HG expression and overall survival (OS) across the different cohorts. A positive relationship between MIR155HG expression levels and the survival rates was observed (Fig. 1C), which was also supported in the Kaplan–Meier (KM) plotter (Fig. 1D). To further interrogate the relationship between MIR155HG levels and the prognosis of LUAD patients, we established a MIR155HG-related gene signature based on the top 100 genes (Table S2) of correlation with MIR155HG in the TCGA-LUAD. Patients in the multiple lung adenocarcinoma cohorts were divided into two groups according to the MIR155HG-related gene signature, and OS was significantly higher in the high MIR155HG-related gene signature groups than in the low groups for many cohorts (Fig. S1B). To identify the gene signatures and pathways associated with MIR155HG expression, we used Gene Ontology Enrichment Analysis (GO) on MIR155HG-related gene signature, which showed a significant enrichment in immune-related signaling including regulation of T cell activation (Fig. 1E). We speculated that the controversial behavior of MIR155HG between the tumor intrinsic oncogenic role and favorable prognosis factor could be related to the immune environment.

In order to understand the role of the immune system affecting the performance of MIR155HG, we inoculated MIR155HG overexpression, knockdown, or control cells in either immunodeficient or PBMC-transferred immunodeficient NCG mice [16] (Fig. 1F). The efficiency of constructed sh-MIR155HG and overexpression plasmids were confirmed in vitro, respectively (Fig. S1C, D). We observed significant changes in tumor growth in immunodeficient mice between MIR155HG overexpression and the control group or MIR155HG knockdown and the control (Figs. 1G, H and S1E), which supported the tumor intrinsic oncogenic role of MIR155HG. Meanwhile, no significant changes in tumor growth in PBMC-transferred NCG mice were observed perturbing the MIR155HG expression (Figs. 1I, J and S1F). Taken together, these results indicate that the existence of immune system abrogates tumor intrinsic oncogenic role of MIR155HG in lung adenocarcinoma.

### MIR155HG is positively associated with CD8^+^ T cells infiltrating

**Fig. 2:**
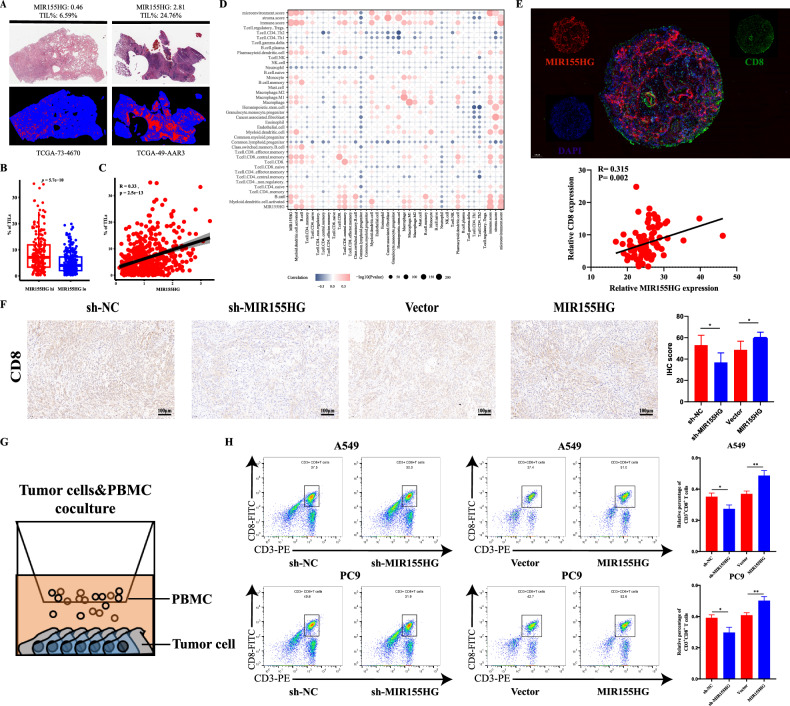
MIR155HG is positively associated with CD8^+^ T cells infiltrating in vivo and in vitro. **A** Representative H&E staining and computational staining images of LUAD from TCGA, which were retrieved from the CANCER Digital Slide Archive and TCIA, respectively. Normalized MIR155HG expression and TIL percentage values are shown above corresponding images. **B** Quantification of estimated TIL proportions in MIR155HG^hi^ and MIR155HG^lo^ LUAD patients. **C** Correlations of MIR155HG levels with the percentages of TILs in LUAD tissues. **D** Correlations of normalized MIR155HG expression with predicted immune cell fractions in LUAD. **E** Immunofluorescence in 94 LUAD patient tissue microarrays and correlations between MIR155HG and CD8^+^ T cells infiltration. **F** Representative images and quantification of CD8 IHC staining in NCG mice tumors with MIR155HG knockdown or overexpression; Scale bar, 100 μm. **G** Schematic view of T cell chemotaxis assay. **H** CD3^+^CD8^+^ T cells in A549 and PC9 cells with MIR155HG knockdown or overexpression detected flow cytometry analysis. Results are presented as mean ± SEM, *n* = 3. *p < 0.05, **p < 0.01.

To further elucidate the relationship between MIR155HG expression and tumor immune profiles, we applied the convolutional neural network–based atlas developed by The Cancer Image Archive (TCIA) [17], which found that the estimated proportion of tumor-infiltrating lymphocytes (TILs) was positively associated with MIR155HG levels in human LUAD (Fig. 2A–C). Next, we used the CIBERSORTx algorithm, which deconvolved the genomic data to estimate the fraction of immune cells in tumor tissues, and we generated correlations between MIR155HG expression and the proportion of the immune cell population. The results showed that MIR155HG expression levels in tumors were positively correlated with multiple antitumoral immune cell types, including CD8^+^ T cells, B cells and dendritic cells (Fig. 2D). To systematically depict the TME between MIR155HG and LUAD, we evaluate the association between MIR155HG expression level and major immune cell subtypes by various algorithms, including Mcp-counter, Quantiseq, Timer and Xcell. The correlation between MIR155HG expression levels and CD8^+^ T cells remained statistically significant across all algorithms used (Fig. S2A). Furtherly, we applied immunofluorescence to analyze the expression of MIR155HG and CD8^+^ T cell infiltration in 94 LUAD patient tissue microarrays and found that MIR155HG was positively correlated with CD8^+^ T cell infiltration (Fig. 2E). Using immunohistochemistry, we found that infiltrated CD8^+^ T cells were reduced in the sh-MIR155HG group and increased in the overexpression group (Fig. 2F), respectively. Together, it can be inferred that elevated MIR155HG expression is positively related to increased CD8^+^ T cells infiltration in lung adenocarcinoma. To further verify whether MIR155HG could recruit CD8^+^ T cells, we employed an in vitro T cell migration assay, co-culturing activated PBMC from healthy donors with human A549 cell lines (Fig. 2G). The results showed that the sh-MIR155HG group recruited fewer CD8^+^ T cells than the sh-NC group, while the overexpression group recruited more (Fig. 2H). Collectively, these results indicate that MIR155HG promotes the recruitment of CD8^+^ T cells both in vivo and in vitro.

### MIR155HG regulates CCL5 expression in LUAD

**Fig. 3:**
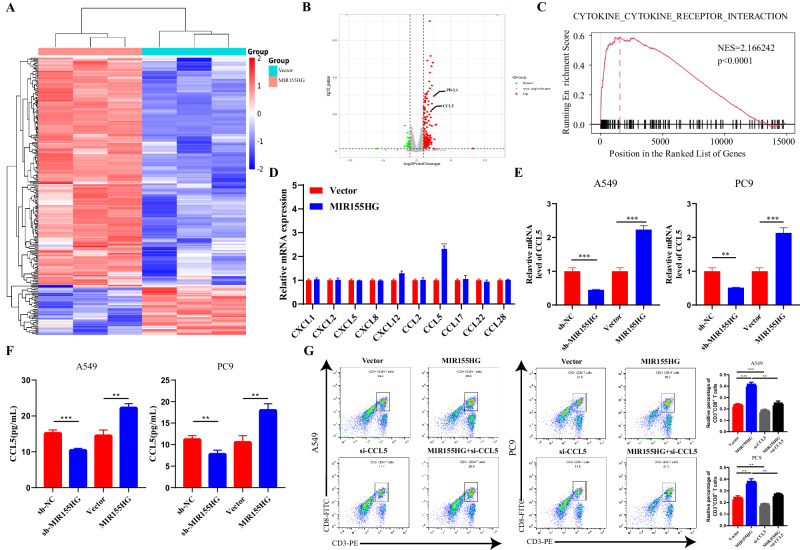
MIR155HG regulates CCL5 expression in LUAD. **A** Heatmap of upregulated/downregulated genes in MIR155G-overexpressed A549 cells compared with parental A549 cells by RNA-sequence. **B** Volcano plot for the DEGs in MIR155G-overexpressed versus vector A549 cells. **C** GSEA plots of Cytokine-cytokine receptor interaction pathway-related signatures. **D** mRNA levels of several chemokine in Vector and MIR155HG-transfected A549 cells by qRT-PCR. **E** mRNA levels of CCL5 in Vector and MIR155HG-transfected A549 and PC9 cells by qRT-PCR. **F** ELISA detection of CCL5 in A549 and PC9 cells with MIR155HG knockdown or overexpressed. **G** Increased recruitment of CD3^+^CD8^+^ T cells in MIR155HG-overexpressing A549 and PC9 cells was abolished by CCL5 knockdown by flow cytometry analysis. Results are presented as mean ± SEM, *n* = 3. **p < 0.01, ***p < 0.001.

To elucidate the potential molecular mechanisms of MIR155HG-regulated recruitment of CD8^+^ T cells in LUAD, we measured gene expression profiling in MIR155HG-overexpressed A549 cells and the control (Fig. 3A). Hierarchical clustering revealed significantly altered expressions of 67 genes (fold change >1.50) in MIR155HG-overexpressed A549 cells compared with the control cells (Fig. 3B and Table S3). To identify the gene pathways associated with MIR155HG expression, we used gene set enrichment analysis (GSEA) and found that cytokine-cytokine receptor interaction was significantly enriched in MIR155HG-overxpressed A549 cells compared to the control (Fig. 3C). Several chemokines previously reported to be associated with the recruitment of CD8^+^ T cells [18, 19] were overlapped with significant differentially expressed genes (DEGs) mentioned above resulting in 10 candidate genes, among which only CCL5 expression increased corresponding to MIR155HG overexpression (Fig. 3D). The variant mRNA and protein levels of CCL5 were detected through qRT-PCR and ELISA assays, respectively, which revealed the similar trends among CCL5 and MIR155HG (Fig. 3E, F).

Subsequently, we conducted a T cell migration assay to further determine the function of CCL5, which uncovered that knockdown of CCL5 mitigated the recruitment effect of MIR155HG on CD8^+^ T cells (Fig. 3G). We also performed a T cell migration assay to determine whether the recruitment effect of MIR155HG depends on miR-155 for that previous studies have reported that MIR155HG relies on miR-155 for its biological functions [20]. The results of our study showed that the miR-155 inhibitor had no effect on the recruitment effect of MIR155HG on CD8^+^ T cells (Fig. S2B, C). These data suggest that MIR155HG promotes the recruitment of CD8^+^ T cells through the regulation of chemokine CCL5.

### MIR155HG interacts with YBX1 protein to increase the protein stability

**Fig. 4:**
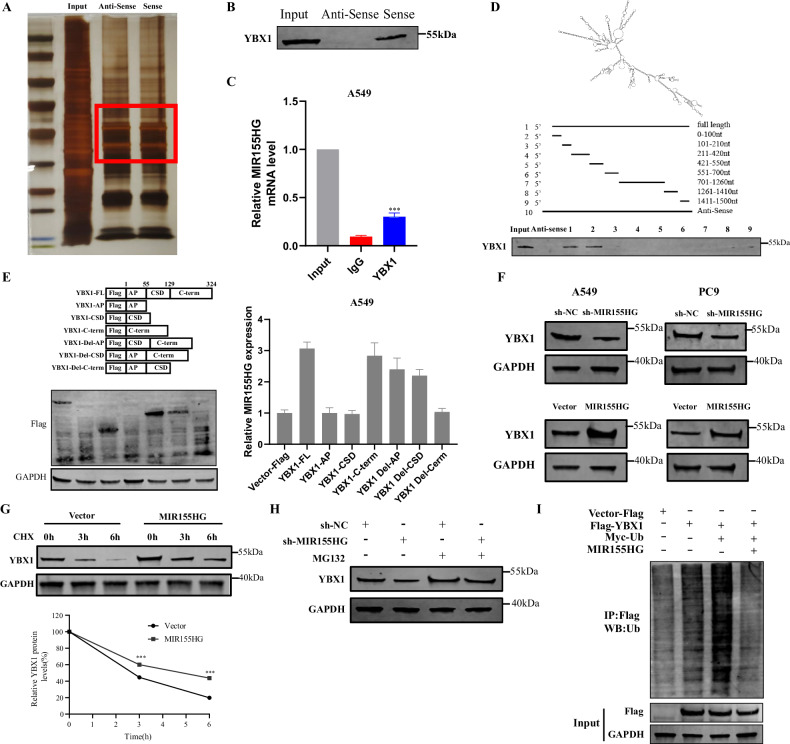
MIR155HG interacts with YBX1 protein to increase the protein stability. **A** Proteins retrieved from the MIR155HG pull-down assay were analyzed by SDS-PAGE. **B** Western blot analysis of the proteins retrieved from the MIR155HG pull-down assay using an anti-YBX1 antibody. **C** RIP assays using an anti-YBX1 antibody showed that YBX1 interacts with MIR155HG in A549 cells by qRT-PCR. **D** Immunoblot detection of the YBX1 protein in A549 cells as retrieved by in vitro transcribed biotinylated RNAs of different constructs of MIR155HG or its antisense sequence (negative control). **E** RIP assays were performed using an anti-Flag antibody in A549 cells transfected with different constructs of YBX1. qRT-PCR was used to measure the enrichment of MIR155HG. Western blot was used to evaluate the expression of different constructs of YBX1. **F** Western blot analysis of YBX1 and GAPDH in A549 and PC9 cells with MIR155HG knockdown or overexpressed. **G** The protein levels of YBX1 were measured in MIR155HG-overexpressing A549 cells by western blot. Cells were treated with CHX (50 mg/mL) for 3 or 6 h before harvest. **H** The protein levels of YBX1 were checked in sh-MIR155HG A549 cells by western blot. Cells were treated with MG132 (20 mmol/L) for 3 h before harvest. **I** A549 cells were transfected with Flag-YBX1, Myc-Ub, or MIR155HG plasmid and treated with MG132 (20 mmol/L) for 3 hours. The ubiquitinylated YBX1 was measured by western blot using an anti-Ub antibody following the immunoprecipitation of Flag-PKM2 with an anti-flag antibody. Results are presented as mean ± SEM, *n* = 3. ***p < 0.001.

To characterize the molecular mechanism of MIR155HG in LUAD, we first determined the subcellular location of MIR155HG detected by nucleocytoplasmic separation and RNA fluorescence in situ hybridization (FISH) assays. The results confirmed that MIR155HG was mainly located in the cytoplasm (Fig. S3A, B). LncRNA localization in the cytoplasm can enable interactions with different functional protein partners and targets of action [21]. To further identify targets directly regulated by MIR155HG, we performed an RNA pull-down assay to isolate proteins that specifically bind to MIR155HG (Fig. 4A). The retrieved proteins were subjected to SDS-PAGE electrophoresis analysis, and several additional differential bands were selected for a mass spectrum analysis. Based on the functional annotation of the proteins detected by mass spectrometry, we identified YBX1 as a putative MIR155HG-associated protein among the highly abundant proteins (Fig. S3C). Western blotting further confirmed the binding of MIR155HG to YBX1 using the retrieved proteins in the RNA pull-down assay (Fig. 4B). RIP assays performed with an anti-YBX1 antibody also demonstrated the interaction between YBX1 and MIR155HG (Fig. 4C). To identify which region of MIR155HG binds to YBX1, we constructed a series of MIR155HG truncation based on the predicted secondary structure of MIR155HG in the RNAfold database (http://rna.tbi.univie.ac.at//cgi-bin/RNAWebSuite/RNAfold.cgi) (Fig. 4D) RNA fragments were transcribed in vitro from these truncation constructs and employed in RNA pull-down assays. We performed immunoblotting analysis of YBX1 in protein samples isolated by these different MIR155HG constructs, and found that the RNA fragments with the 0–100 truncation nearly completely lost their ability to bind YBX1, indicating that this region is essential for MIR155HG to bind to YBX1 (Fig. 4D). This result was consistent with predicted potential YBX1-binding region of MIR155HG (0–100) using the catRAPID omics tool (http://s.tartaglialab.com/page/catrapid_omics_group) (Fig. S3D). Additionally, to investigate which domain of YBX1 is involved in its interaction with MIR155HG, we constructed a series of Flag-tagged YBX1 truncation plasmids according to the protein structure of YBX1 [22] and verified the truncation with WB (Fig. 4E left). Then we conducted RIP assays to indicate that the C-terminal region (129–324) of YBX1 is essential for its binding to MIR155HG (Fig. 4E right).

We also measured the transcriptional and translational levels of YBX1 in MIR155HG-knockdown or MIR155HG-overexpressing lung cancer cells, and no significant changes in the mRNA levels of YBX1 were observed when compared to the corresponding controls (Fig. S3E), while the protein levels of YBX1 were significantly decreased in MIR155HG-knockdown cells and increased in MIR155HG-overexpressing cells (Figs. 4F and S3F). It suggested that MIR155HG can increase the levels of YBX1 protein at the post-transcriptional level. To further validate this observation, we used the protein synthesis inhibitor cycloheximide (CHX) to evaluate the effect of MIR155HG on the degradation of YBX1. MIR155HG overexpression in A549 cells extended the half-life of YBX1 (Fig. 4G). Moreover, we observed that inhibiting proteasome activity prevented sh-MIR155HG–induced endogenous YBX1 downregulation in A549 cells (Fig. 4H), suggesting that YBX1 degradation via the ubiquitin-proteasome pathway could be inhibited by MIR155HG. To confirm these data, we evaluated the effect of MIR155HG on YBX1 ubiquitination in lung cancer cells (Fig. 4I), which showed that MIR155HG expression could increase the stability of YBX1 via reducing its ubiquitous modification. Together, these results demonstrate that MIR155HG increases the protein stability of YBX1 by dampening its ubiquitin.

### MIR155HG promotes CCL5 transcription through YBX1 in LUAD

**Fig. 5:**
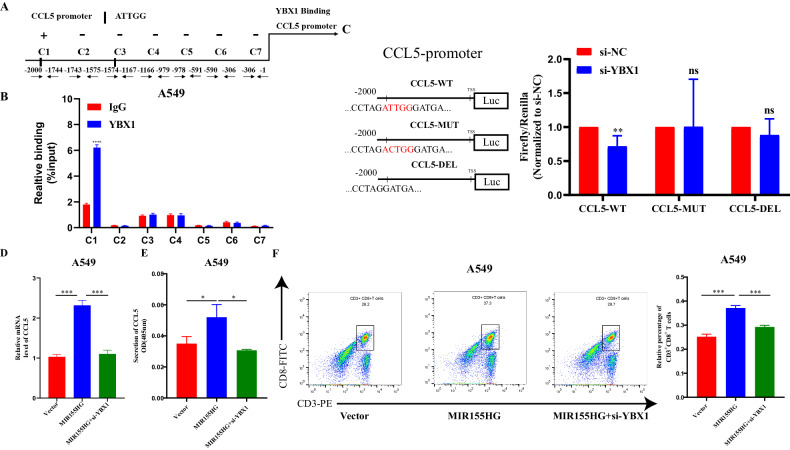
MIR155HG promotes CCL5 transcription through YBX1 in LUAD. **A** The human CCL5 proximal promoter (−1 to −2 kb) was divided into seven different segments. **B** ChIP analysis was used to study the potential binding of YBX1 in A549 cells. YBX1 bound to the designated regions (C1, containing the Y-box sequence). ChIP assays were performed using a YBX1-specific antibody, R-IgG was the ChIP control. **C** Three different luciferase vectors constructed according to CCL5 promoter C1 region were co-transfected with si-YBX1 into A549 cells. The relative firefly/Renilla luciferase activities were analyzed in the cells 24 h after transfection. **D** Increased CCL5 expression in MIR155HG-overexpressing A549 cells was abolished by YBX1 knockdown by qRT-PCR. **E** Increased secretion levels of CCL5 in MIR155HG-overexpressing A549 cells were abolished by YBX1 knockdown by ELISA assay. **F** Increased recruitment of CD3^+^CD8^+^ T cells in MIR155HG-overexpressing A549 cells was abolished by YBX1 knockdown by flow cytometry analysis. Results are presented as mean ± SEM, *n* = 3. *p < 0.05, **p < 0.01, ***p < 0.001.

YBX1, as a transcription factor, plays an important role in regulating genes in cancer [23, 24]. Therefore, we investigated whether YBX1 could regulate CCL5 transcription. YBX1 binds to the Y-box sequence (ATTGG) in the promoter of target genes [25]. We searched the proximal promoter region of human CCL5 (−1 to −2 kb upstream of the transcription start site (TSS)) and found two potential Y-box sequence (Fig. 5A). To confirm its relevance, we performed ChIP assays in A549 cells. As shown in Fig. 5B, YBX1 bound to the region of the CCL5 promoter containing the Y-box (C1). To verify the ChIP-PCR assay results, we constructed wildtype, mutation and deletion luciferase plasmids according to the C1 region respectively (Fig. 5C left and Fig. S3G) and then transfected these plasmids along with YBX1 siRNA in A549 cells, which showed that luciferase activity was reduced in the luciferase plasmid containing the Y-box sequence with knockdown of YBX1. However, knockdown of YBX1 had no effect on luciferase activity in luciferase plasmid lacked the Y-box or contained a Y-box mutation (Fig. 5C right). Additional, knockdown of YBX1 reversed the mRNA and secretion levels of CCL5 upregulated by MIR155HG in A549 cells (Fig. 5D, E). Finally, we conducted a T cell migration assay to further determine the dependency of YBX1 and found that knockdown of YBX1 reversed the recruitment effect of MIR155HG on CD8^+^ T cells (Fig. 5F).

### MIR155HG upregulates PD-L1 transcription to hamper the activity of recruited CD8^+^ T cells

**Fig. 6:**
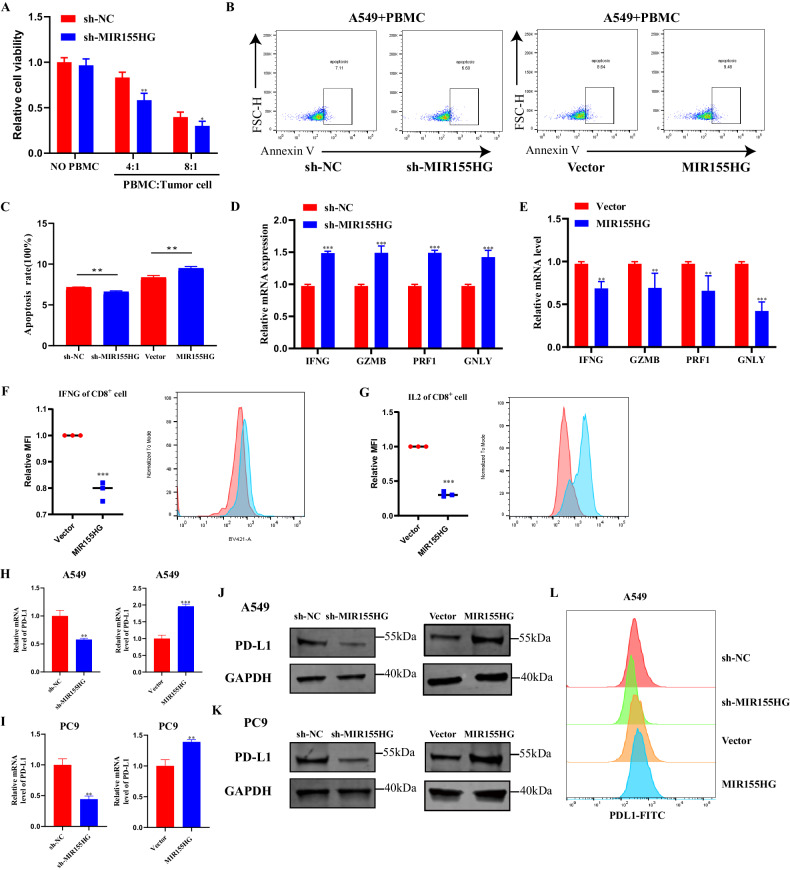
MIR155HG upregulates PD-L1 transcription to hamper the activity of recruited CD8^+^ T cells. **A** CCK8 assay detected the killing of tumor cells by indicated activated PBMC. A549 cells were co-cultured with/without PBMC for 24 h. **B** Activated PBMC were co-cultured with A549 cells with MIR155HG knockdown or overexpressed for 24 h. The PBMC were collected and stained with FITC-Annexin V, then subjected to flow cytometry analysis. **C** Quantification of apoptosis cells detected by flow cytometry. **D** qRT-PCR was performed to detect PRF1, GZMB, GNLY and IFNG in activated PBMC co-cultured with A549- sh-NC/sh-MIR155HG and A549- Vector /MIR155HG (**E**) for 48 h. **F** Flow cytometry analysis detected the intracellular IFNG level and IL-2 level (**G**) of CD3^+^CD8^+^ T cells in PBMC after co-culturing with A549-vector/MIR155HG cells for 3 days. **H** qRT-PCR analysis of PD-L1 mRNA in A549 cells and PC9 cells (**I**) with MIR155HG knockdown or overexpressed. **J** Western blot analysis of PD-L1 and GAPDH in A549 cells and PC9 cells (**K**) with MIR155HG knockdown or overexpressed. **L** Flow cytometry analysis detected PD-L1 in A549 cells with MIR155HG knockdown or overexpressed. Results are presented as mean ± SEM, *n* = 3. **p < 0.01, ***p < 0.001.

In our study, MIR155HG was found to promote the recruitment of CD8^+^ T cells. To further test its role in regulating antitumor T cell immunity, we performed T cell-mediated killing assay in vitro by employing a co-culture system in which activated PBMC from healthy donors were co-cultured with human A549 cell lines. A549 cells with MIR155HG knockdown was more vulnerable against T cell killing (Fig. 6A). The number of apoptotic cells was higher in MIR155HG-knockdown cells co-cultured with PBMCs compared to those co-cultured with control A549 cells, suggesting that the suppressive function of tumor cells on T cells was attenuated by MIR155HG knockdown (Fig. 6B, C). In addition, the mRNA levels of PRF1 (Perforin 1), GZMB (Granzyme B), GNLY (Granulysin), IFNG (Interferon gamma) in PBMCs were increased upon co-culturing with MIR155HG knockdown, and decreased upon co-culturing with MIR155HG overexpression (Fig. 6D, E). Flow cytometry analyses showed increased IFN-gamma and Interleukin 2 (IL-2) levels in CD8^+^ T cells co-cultured with MIR155HG knockdown compared to corresponding control cells (Fig. 6F, G). Taken together, these data suggested that the overexpression of MIR155HG in tumor cells suppresses T cell-mediated antitumor activity.

Our exploration of TCGA-LUAD data and PBMC from patients also suggested a relationship between MIR155HG expression and immune checkpoints, such as PD-L1 and PD-1 (Fig. S4A–C). PD-L1 on tumor cells binds to PD-1 on activating T cells, leading to the exhaustion and apoptosis of T cells subsequently, which has been identified as a key process in tumor cell-mediated immune escape [26]. As PD-L1 was significantly different in the previous RNA-seq results (Fig. 3A, B), we explored the association between PD-L1 and MIR155HG by qRT-PCR and Western Blot assays. While MIR155HG was knockdown or overexpressed, we found that mRNA and protein levels of PD-L1 were obviously increased after overexpression of MIR155HG, while knockdown of MIR155HG had the opposite result (Figs. 6H–K and S4D). This result was further validated by flow cytometry analysis (Fig. 6L). Meanwhile, we observed that anti-PD-L1 mAb could reverse the expression of TOX of T cell, a maker of T cell exhaustion, resulting from overexpression of MIR155HG (Fig. S4E).

Previous study shows that YBX1 transcriptionally regulates PD-L1 expression in doxorubicin-resistant HepG2 cells [27] Therefore, we searched the proximal promoter region of human PD-L1 (−1 to −1.5 kb upstream of the transcription start site (TSS)) and found a Y-box sequence (Fig. S4F). To validate its relevance in lung tumor cells, we performed ChIP assays using A549 cells. As shown in Fig. S4G, YBX1 bound to the region of the PD-L1 promoter containing the Y-box (P6). To confirm the ChIP-PCR assay results, we constructed wildtype, mutation and deletion luciferase plasmids according to the P6 region (Fig. S4H left) and then transfected these plasmids along with YBX1 siRNA in A549 cells. The results shown that luciferase activity was reduced in the luciferase plasmid containing the Y-box sequence with knockdown of YBX1. However, knockdown of YBX1 had no effect on luciferase activity in luciferase plasmid lacked the Y-box or contained a Y-box mutation (Fig. S4H right). Collectively, these results indicate that MIR155HG suppresses antitumor T cell immunity by promoting PD-L1 transcription through YBX1.

### MIR155HG correlates with the efficacy of PD-L1 mAb therapy in LUAD patients and improves antitumor effect of PD-L1 blockade in vivo

**Fig. 7:**
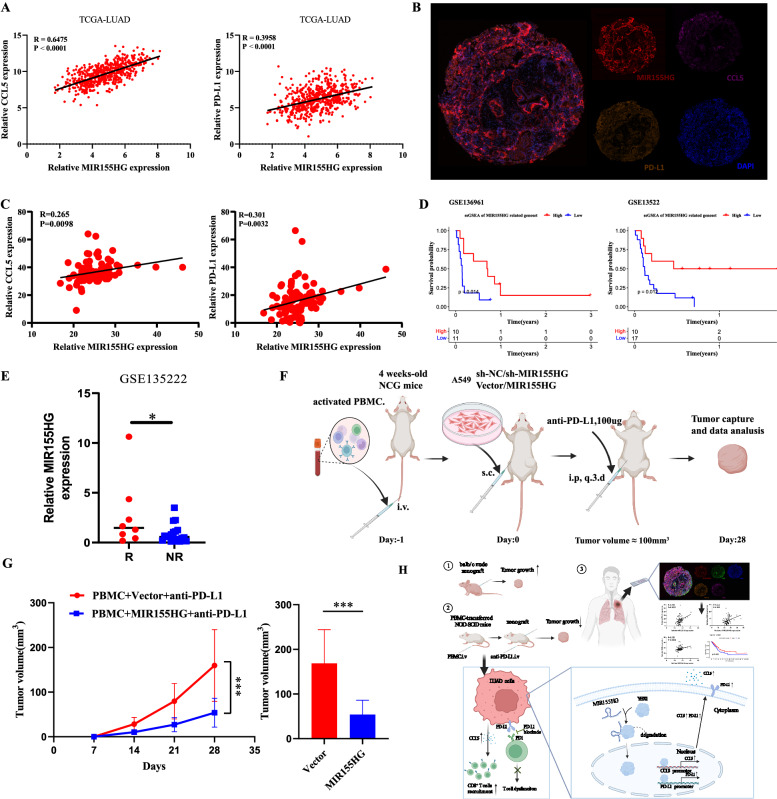
MIR155HG correlates with the efficacy of PD-L1 mAb therapy in LUAD patients and improves antitumor effect of PD-L1 blockade in vivo. **A** Correlations of MIR155HG expression with CCL5 and PD-L1 expression in LUAD from TCGA database. **B** Representative images of immunofluorescence in 94 LUAD patient tissue microarrays and correlations between MIR155HG, CCL5 and PD-L1. **C** Correlations of MIR155HG expression with CCL5 and PD-L1 expression in microarrays. **D** Kaplan–Meier survival curves of survival probability according to MIR155HG-related gene signature in LUAD cohorts (GSE136961, GSE13522). **E** Relative MIR155HG expression between response and non-response in GSE135222. **F** Schematic view of mice model in PBMC-transferred NCG mice with PD-L1 mAb treatment. **G** Tumor growth curves and tumor volumes of Vector and MIR155HG-transfected A549 cells with PD-L1 mAb treatment in PBMC-transferred NCG mice. **H** Graphic abstract. Results are presented as mean ± SEM, *n* = 5. *p < 0.05, ***p < 0.001.

We first explored the TCGA-LUAD dataset and found that MIR155HG was positively correlated with PD-L1 and CCL5 expression respectively (Fig. 7A). Meanwhile, to establish the correlation between MIR155HG, PD-L1 and CCL5 in LUAD patients, we applied immunofluorescence to analyze the expression of MIR155HG, PD-L1 and CCL5 in 94 LUAD patients tissue microarrays (Fig. 7B), which also revealed that MIR155HG expression is positively associated with PD-L1 and CCL5 expression in LUAD microarray (Fig. 7C). The Immunophenotypic Score (IPS) is a score that integrates the scores for four distinct immune phenotypes (antigen-presenting cells, effector cells, suppressive cells, and checkpoints) using a computational method to derive an aggregate immune-related score (Fig. S5A). Higher IPS scores have been associated with a more favorable response to immunotherapy [28]. We found that patients with higher MIR155HG expression had correspondingly higher immunophenotypic scores (Fig. S5B), suggesting a potentially effective response to immunotherapy. Furtherly, survival analysis was performed in the lung adenocarcinoma patients involved in the published available datasets (GSE136991 and GSE135222) containing immunotherapy responses based on the established MIR155HG-related gene signature. The results showed that MIR155HG-related gene signature was positively associated with the longer survival (Fig. 7D). Meanwhile, our results showed that patients with immunotherapy response had a higher expression of MIR155HG than those patients with non-response in the LUAD cohort GSE135222(Fig. 7E), suggesting a potentially biomarker for predicting the efficacy of immunotherapy.

To investigate whether overexpression of MIR155HG enhanced the therapeutic effect of PD-L1 mAb treatment, we utilized anti-human PD-L1 mAb to treat PBMC-transferred immunodeficient NCG mice inoculated subcutaneous xenograft with A549-vector/MIR155HG or A549-sh-NC/sh-MIR155HG lung cancer cells (Fig. 7F). We observed that the sh-MIR155HG group increased the tumor volume compared with the sh-NC group (Fig. S5C, D). However, PD-L1 mAb treatment significantly decreased the tumor volume of MIR155HG overexpression group compared with the control (Figs. 7G and S5E). Together, these results suggested that MIR155HG can serve as a biomarker for predicting the efficacy of immunotherapy in LUAD and have a synergistic effect with PD-L1 mAb treatment in preclinical animal studies.

## Discussion

Our study revealed that MIR155HG abrogates its intrinsic oncogenic role by inducing tumor CCL5 secretion dependent on YBX1, thus promoting the recruitment of CD8^+^ T cells. In addition, the combination of MIR155HG overexpression and PD-L1 mAb can increase the efficacy of PD-L1 mAb validated by mouse models. Therefore, we have unraveled the molecular mechanism of MIR155HG regulation in tumors and discovered a biomarker of immunotherapy efficacy in lung adenocarcinoma and a combination treatment strategy (Fig. 7H).

MIR155HG acts as an oncogene that propels malignant progression in various cancers [29]. Supporting previous studies, our work revealed that MIR155HG indeed fosters malignant progression in balb/c nude mice. However, a positive correlation between MIR155HG and overall survival in TCGA and multiple LUAD cohorts was noted. Recently, there has been growing interest in the influence of genes on the immune microenvironment [30], and several studies have highlighted the differential roles genes play in immune-complete and immune-deficient environments [31, 32]. To delve deeper into the role of MIR155HG, we examined its functionality in subcutaneous tumor-bearing PBMC-transferred NCG mice. Unexpectedly, MIR155HG no longer presented its cancer-promoting abilities in these mice, suggesting that the immune microenvironment restricts its oncogenic function. This finding implied a potential role for MIR155HG in modulating the microenvironment, challenging existing perceptions and prompting us to select MIR155HG for further investigation. MIR155HG, classified as the primiRNA of miR-155, is reported to be associated with immune infiltration and prognosis of breast cancer [33]. Subsequent experiments revealed that after MIR155HG overexpression, inhibiting miR-155 did not affect its capacity to recruit CD8^+^ T cells, which revealed that MIR155HG’s function of our study is independent of miR-155, motivating the continued exploration of MIR155HG’s molecular mechanisms.

CD8^+^ T cells, as a key immune cell population, play a significant role in the immune microenvironment [34]. A correlation has been established by various studies between the degree of CD8^+^ T cell infiltration within the immune microenvironment and the predicted efficacy of immunotherapy [35, 36]. T cells can also be summoned by a wide array of cytokines. In our research, we discovered that MIR155HG can recruit more CD8^+^ T cells by CCL5, both in vivo and in vitro, through immunohistochemistry and T cell recruitment assay, thereby mitigating its own cancer-promoting effects. Interestingly, while the recruitment of additional CD8^+^ T cells was noted, the cytolytic activity of these cells was decreased, ostensibly due to elevated PD-L1 expression. We hypothesized that this could be an inherent cellular mechanism to curb an excessive immune response through the upregulation of PD-L1, thereby maintaining immune homeostasis [37]. In a mouse model augmented with PD-L1 monoclonal antibodies, it was evident that tumors in the MIR155HG knockdown group proliferated, while those in the MIR155HG-overexpressing group, treated with PD-L1 mAb, significantly regressed. This emphasizes that MIR155HG had a profound impact on CD8^+^ T cell recruitment, rather than merely promoting PD-L1, that led to functional compromise of T cells. We were also able to demonstrate that the depletion of MIR155HG on T cells could be counteracted with PD-L1 monoclonal antibodies.

Previous studies have reported that lncRNA localization in the cytoplasm can enable interactions with different functional protein partners and targets of action [21]. In this study, we observed that MIR155HG is primarily located in the cytoplasm, which interacts with the YBX1 protein. Our results showed that MIR155HG binds to YBX1 in the cytoplasm, thereby increasing the protein stability of YBX1 and promoting CCL5 transcription. Knockdown of YBX1 reduces the CCL5 expression and thus decreases the recruitment of CD8+ T cells, all of which indicate that YBX1 plays a crucial role in mediating the interaction of MIR155HG with CD8^+^ T cells. Additional research is required to identify the specific components implicated in the ubiquitination of YBX1.

In LUAD patient tumor samples, we confirmed a positive correlation between MIR155HG, CCL5, CD8, and PD-L1 expression. Additionally, we found that MIR155HG is associated with IPS in the TCGA database. By assessing the clinical significance of MIR155HG in PD-L1 mAb-treated LUAD patients, we found that high MIR155HG expression indicated a better therapeutic outcome and longer OS times of LUAD patients treated with PD-L1 mAb in two cohorts, further indicating its potential as a biomarker for predicting immunotherapy efficacy. The use of a larger cohort of immunotherapy patients with lung adenocarcinoma could further clarify the reliability of the biomarker. Furthermore, in our preclinical animal studies, we found a synergistic effect of MIR155HG overexpression with PD-L1 monoclonal therapy with a significant reduction in tumor volume and extended survival time compared to the control group. Thus, our findings suggest the feasibility of a combination treatment strategy for lung cancer patients. Unfortunately, due to the non-conserved form of MIR155HG, we can only perform this step in the mouse model of PBMC-transferred NCG mouse, suggesting the limitation of our models. The future application of single-cell sequencing technology and humanized mice will enable us to further clarify the role of MIR155HG in the immune microenvironment.

In summary, our study revealed that genes may assume varied roles in immunocomplete and immunodeficient environments. MIR155HG affects the expression of downstream genes CCL5 and PD-L1 by elevating the protein stability of YBX1 and reducing its ubiquitination. MIR155HG serves as a biomarker for predicting the efficacy of immunotherapy in LUAD and it also exhibits a synergistic effect with PD-L1 mAb treatment in preclinical animal studies. Therefore, it is worthwhile to develop further research and clinical translation of MIR155HG.

## Materials and methods

### Cell lines and cell culture

LUAD cell lines (A549 and PC9) were obtained from the American Type Culture Collection (ATCC). A549 cells were cultured in RPMI-1640(KeyGen, China) supplemented with 10% fetal bovine serum (FBS; Life Technologies) while PC9 cells were maintained in DMEM (KeyGen, China). Cycloheximide (CHX, protein synthesis inhibitor) (Selleck, USA) and MG132 (proteasome inhibitor) (Selleck, USA) were used at a final concentration of 10 μg/ml and 50 μM, respectively. All cell lines were cultured in a humidified incubator at 37 °C and 5% CO_2_ according to standard protocols. All cell lines were routinely tested for mycoplasma contamination and had a negative result.

### Patient samples

Thirty pairs of LUAD tissues and adjacent normal tissues were used for RNA extraction for qRT-PCR. Ninety-four LUAD tissues were used for the construction of tissue microarrays (TMAs). All tissue samples were collected in compliance with informed consent policy before surgery. The study was approved by the Ethics Committee of The Affiliated Cancer Hospital of Nanjing Medical University. Samples were obtained from biobank of Jiangsu Cancer Hospital (Jiangsu Institute of Cancer Research & The Affiliated Cancer Hospital of Nanjing Medical University). All patients had signed informed consent for donating their samples.

### shRNA, plasmid and cell transfections

ShRNA targeting MIR155HG and plasmid overexpressing MIR155HG were purchased from RiboBio, China and shRNA targeting MIR155HG, respectively. The siRNAs targeting CCL5 and YBX1 were purchased from RiboBio. All sequences are listed in the Supplementary table. Cells with 80% confluence were transfected using Lipofectamin RNAiMAX (Invitrogen, USA) and Lipofectamine 3000 (Invitrogen, USA) and the experimental procedure was according to protocol. Cells were collected or for further experiments 24 h after transfection. The constructions were verified by qRT-PCR. All Primer sets, Sequences of siRNAs and shRNA sets are listed in Table S1.

### RNA extraction, reverse transcription, and quantitative real-time PCR (qRT-PCR)

Total RNA from cells and fresh tissues was isolated using TRIzol reagent (Invitrogen, USA). For reverse transcription, cDNA was synthesized using the PrimeScript RT Reagent Kit (Takara, Japan). The reaction was carried out for 15 min at 37 °C, 5 min at 85 °C, and then 4 °C until further use. qRT-PCR was preformed using ABI QuantStudio6Flex (Thermo, USA) and ChamQ Universal SYBR Green PCR kit (Vazyme, China). GAPDH, Actin, and U6 were used as the internal controls. The relative level of each target RNA was calculated using the 2^△△Ct^ method and normalized to the internal controls.

### Western blotting and ELISA assay

Total protein was extracted from cells lysed on ice with radioimmunoprecipitation assay (RIPA) lysis buffer (Thermo, USA) and Protease Inhibitor Cocktail (Roche, USA). BCA Protein Assay Kit (KeyGEN, China) was used to determine the protein concentration. Equal amounts of protein were resolved on sodium dodecyl sulfate-polyacrylamide gels electrophoresis (SDS-PAGE) and transferred to a PVDF membrane (Millipore, USA). The membranes were blocked in 5% non-fat milk and incubated with primary antibodies at 4 °C overnight, followed by incubation with corresponding secondary antibodies. The immunoblots were detected using a grayscale ratio with the Odyssey CLx Imaging System (LI-COR, USA). Primary antibodies against YBX1(20339-1-AP, dilution:1:5000), PD-L1(28076-1-AP, dilution:1:300) and GAPDH (60004-1-Ig, dilution:1:50,000) were bought from proteintech. Human CCL5 levels in conditioned medium from MIR155HG-sh/MIR155HG-overexpressed A549 cells were measured by ELISA using the Human CCL5 ELISA kit (Fcmacs, China) according to the manufacturer’s protocol.

### Isolation and activation of peripheral blood mononuclear cells (PBMC)

The Peripheral Blood Mononuclear Cells (PBMC) were isolated from the peripheral blood in accordance with the manufacturer’s protocol using a PBMC separation tube (KeyGen, China). The process began with the careful addition of diluted blood to the top of the BMC separation tube. Subsequent to centrifugation, the mononuclear cell was isolated and given a solitary wash with PBS. Characteristically, 4 × 10^6^ PBMC were activated with a 20 µl T Cell Activation/Expansion Kit (Miltenyi Biotec, USA) in a 1 ml T cell serum-free medium (Fcmacs, China). This medium was also supplemented with 20IL/mL IL-2 (R&D, USA). In the interests of further expansion, viable cell density was routinely adjusted to 1 × 10^6^ cells/ml every two to three days, assisted by the addition of fresh, complete T cell serum-free medium supplemented again with 20IL/mL IL-2.

### Mouse xenograft models

All animal experiments were conducted in conformity with the Institutional Animal Care and Use Committee of Nanjing Medical University provided full approval for this research. BALB/c Nude mice were acquired from GemPharmatech, Inc. (Nanjing, China) and randomly divided into two groups (*n* = 5, no blinding was performed, respectively). Cells were injected subcutaneously into the axilla of nude mice (5 × 10^6^ cells/mouse). Adult female NCG mice (4 weeks) were purchased from GemPharmatech (Nanjing, China) and randomly assigned into experiment groups. A549 tumor cells (sh-NC/sh-MIR155HG, Vector/MIR155HG) of 5 × 10^6^ were injected into the right flank of NCG mice. Tumor volume was calculated by the formula: volume = *ab*^2^/2 (a, the longer axis; b, the shorter axis). As previously described, PBMC from healthy donors underwent activation and expansion. A day before the tumor cell injection, PBMC was translocated adoptively into the NCG mice through the tail vein, with each mouse receiving 1 × 10^7^ cells intravenously. Following implantation, the tumor’s maximum diameter underwent measurement every two days.

### Immunohistochemistry

The formalin‐fixed paraffin‐embedded mice tumor tissue specimens were cut as serial 5‐μm sections. The detailed steps are as previously reported [38]. In brief, the sections were treated with the primary antibodies CD3(Proteintech, China) for an overnight period. The sections were then stained with a 3,3-diaminobenzidine solution and treated with an HRP-polymer-conjugated secondary antibody (CST, USA) for 1 h at 37 °C.

### Flow cytometry analysis

For Cell Surface Flow Cytometry Staining, cells were stained with fluorescently labeled antibodies to the surface proteins at a 1:100 dilution in Cell Staining Buffer (BioLegend, USA) for 30 min at 4 °C. For intracellular protein analyses, cells were then fixed and permeabilized by using Fixation Buffer (BioLegend, USA) and Intracellular Staining Permeabilization Wash Buffer (BioLegend, USA) according to the manufacturers’ instructions. Permeabilized cells were then incubated with fluorescently labeled antibodies to the intracellular proteins for 30 min at 4 °C. Flow cytometry analysis (BD FACSCalibur, USA) was used for flow cytometry data acquisition, and data were analyzed with FlowJo software (version 10.5.3). Primary antibodies against CD3-PE(BioLegend, USA980008), CD8-FITC(BioLegend, USA,Cat#980008), PD-L1-FITC(BioLegend, USA, Cat#374509), IFNG-BV421(BioLegend, USA, Cat502531) and IL-2-BV421(BioLegend, USA, Cat#500327).

### T cell chemotaxis assay and T cell killing assay in vitro

The chemotaxis of T cells was assessed using a transwell with a 5 µm pore-sized cell culture insert. A549 cells (sh-NC/sh-MIR155HG, Vector/MIR155HG) were placed in the lower wells, while the activated T cells were seeded in the upper wells. Incubation was performed overnight at 37 °C in a 5% CO_2_ environment. Flow cytometry analysis (BD FACSCalibur, USA) was conducted on the samples after 24 hours.

In the T cell killing assay, activated PBMC was co-cultured with tumor cells. Post-incubation, the tumor cell viability was determined by Cell Counting Kit-8 (CCK8) (KeyGen, China), and tumor cell apoptosis was identified via the Annexin V-FITC Apoptosis Detection Kit (KeyGen, China). The cell viability of adherent tumor cells was gauged using the CCK8 assay as per the manufacturer’s instructions. The results were normalized to the control where no PBMCs were added. These procedures were conducted in triplicate. For apoptosis detection, both suspension cells (predominantly PBMC and dead cells) and adherent cells were gathered and subjected to Annexin V-FITC staining following the manufacturer’s instructions. The samples were analyzed via flow cytometry (BD FACSCalibur, USA).

### Cytoplasmic and nuclear RNA isolation and IF assays

Cytoplasmic and nuclear RNA samples were isolated and purified using an Ambion™ PARIS™ kit (Thermo, USA), in accordance with the manufacturer’s given instructions. To evaluate the expression levels of MIR155HG, GAPDH, and U6 in both cytoplasmic and nuclear compartments, we executed qRT-PCR assay. GAPDH and U6 small nuclear RNA were employed as control measures for cytoplasmic and nuclear samples, respectively.

### RNA pull-down assays

RNA pull-down assays were executed applying the Pierce Magnetic RNA-Protein Pull-Down kit (Thermo Fisher Scientific, USA), adhering to the manufacturer’s stipulations. RNA Polymerase (T7) alongside Biotin RNA Labeling Mix (Roche, USA) were utilized to conduct in vitro transcription, and the transcribed biotinylated RNA (both sense and antisense) underwent purification. Around 8 μg of the purified biotinylated RNA was subsequently combined with magnetic beads, after which, A549 cell lysates were added and incubated overnight at 4 °C. The extracted proteins were then assessed using mass spectrometry and Western blotting.

### RIP

The RNA Immunoprecipitation (RIP) assay was conducted following the manufacturer’s protocols using a MagnaRIP RNA-Binding Protein Immunoprecipitation kit (Millipore, USA). In brief, 5 µg of control IgG antibody and of anti-YBX1 (Proteintech, China) were attached to magnetic beads and incubated with corresponding cell lysates overnight at a temperature of 4 °C. Subsequently, proteinase K was employed to digest the protein, and phenol-chloroform was applied to extract the RNA. Ultimately, the expression of MIR155HG was detected by carrying out reverse transcription and qRT-PCR.

### ChIP

The Chromatin Immunoprecipitation (ChIP) assay was conducted using CHROMATIN IP (CHIP) ASSAY KIT (Millipore, USA). The cells were initially stabilized with 1% formaldehyde and afterward quenched with glycine. Subsequent to this, the cells were resuspended in a lysis buffer and the chromatin solution, which underwent enzymatic digestion, was then immunoprecipitated using an anti-YBX1 (Proteintech, China). The immunoprecipitated DNA was purified by means of spin columns and analyzed via qRT-PCR.

### Dual-luciferase reporter assay

For the CCL5 and PD-L1 luciferase reporter assay, reporter plasmid and corresponding siRNA were co-transfected into A549 cells using Lipofectamine 3000 (Invitrogen Life Technologies, USA) and RNAiMAX(Invitrogen, USA). At 24 h after transfection, cells were analyzed using a Dual-Luciferase Reporter Assay System (Vazyme, China). Firefly luciferase activity was normalized to Renilla luciferase activity.

### Statistical analysis

All results are representative of at least three independent experiments. Statistical analyses and the number of samples (n) were described in detail for each figure. Data are presented as mean ± s.e.m. and computations were processed using GraphPad Prism 8 software. The comparison between two groups utilized either a two-tailed unpaired or paired Student’s t-test. Survival functions were demonstrated by Kaplan–Meier curves with group differences analyzed through a log-rank test. All *P* values are *P < 0.05, **P < 0.01, ***P < 0.001 and ****P < 0.0001.

### Supplementary information

Supplementary Matrials

original western blot

## Data Availability

The mRNA profile and CNV profile were downloaded from UCSC Xena (https://xenabrowser.net/). Kaplan–Meier survival analysis was conducted via Kaplan–Meier Plotter (https://kmplot.com/analysis/). Difference expression between LUAD and normal tissue assay was performed via GEPIA (http://gepia.cancer-pku.cn/). The data supporting the findings of this study are available within the article and its Supplementary Information files and from the corresponding authors on request.

## References

[1] Wang M, Herbst RS, Boshoff C. Toward personalized treatment approaches for non-small-cell lung cancer. Nat Med. 2021;27:1345–56. 34385702 10.1038/s41591-021-01450-2

[2] Yarchoan M, Hopkins A, Jaffee EM. Tumor mutational burden and response rate to PD-1 inhibition. N Engl J Med. 2017;377:2500–1. 29262275 10.1056/NEJMc1713444 PMC6549688

[3] Gandhi L, Rodríguez-Abreu D, Gadgeel S, Esteban E, Felip E, De Angelis F, et al. Pembrolizumab plus chemotherapy in metastatic non-small-cell lung cancer. N Engl J Med. 2018;378:2078–92. 29658856 10.1056/NEJMoa1801005

[4] Wu M, Huang Q, Xie Y, Wu X, Ma H, Zhang Y, et al. Improvement of the anticancer efficacy of PD-1/PD-L1 blockade via combination therapy and PD-L1 regulation. J Hematol Oncol. 2022;15:24. 35279217 10.1186/s13045-022-01242-2 PMC8917703

[5] Park EG, Pyo SJ, Cui Y, Yoon SH, Nam JW. Tumor immune microenvironment lncRNAs. Brief Bioinform. 2022;23:bbab504. 10.1093/bib/bbab504 PMC8769899 34891154

[6] Pitt JM, Marabelle A, Eggermont A, Soria JC, Kroemer G, Zitvogel L. Targeting the tumor microenvironment: removing obstruction to anticancer immune responses and immunotherapy. Ann Oncol. 2016;27:1482–92. 27069014 10.1093/annonc/mdw168

[7] Lei X, Lei Y, Li JK, Du WX, Li RG, Yang J, et al. Immune cells within the tumor microenvironment: Biological functions and roles in cancer immunotherapy. Cancer Lett. 2020;470:126–33. 31730903 10.1016/j.canlet.2019.11.009

[8] Marzagalli M, Ebelt ND, Manuel ER. Unraveling the crosstalk between melanoma and immune cells in the tumor microenvironment. Semin cancer Biol. 2019;59:236–50. 31404607 10.1016/j.semcancer.2019.08.002

[9] Zhang Y, Zhang Z. The history and advances in cancer immunotherapy: understanding the characteristics of tumor-infiltrating immune cells and their therapeutic implications. Cell Mol Immunol. 2020;17:807–21. 32612154 10.1038/s41423-020-0488-6 PMC7395159

[10] Tay C, Tanaka A, Sakaguchi S. Tumor-infiltrating regulatory T cells as targets of cancer immunotherapy. Cancer cell. 2023;41:450–65. 36917950 10.1016/j.ccell.2023.02.014

[11] Lin H, Ni R, Li D, Zhao M, Li Y, Li K, et al. LncRNA MIR155HG overexpression promotes proliferation, migration, and chemoresistance in gastric cancer cells. Int J Med Sci. 2023;20:933–42. 37324190 10.7150/ijms.82216 PMC10266045

[12] Gherzi R, Caratti C, Andraghetti G, Bertolini S, Montemurro A, Sesti G, et al. Direct modulation of insulin receptor protein tyrosine kinase by vanadate and anti-insulin receptor monoclonal antibodies. Biochem Biophys Res Commun. 1988;152:1474–80. 2837189 10.1016/S0006-291X(88)80452-2

[13] He X, Sheng J, Yu W, Wang K, Zhu S, Liu Q. LncRNA MIR155HG promotes temozolomide resistance by activating the Wnt/β-catenin pathway via binding to PTBP1 in glioma. Cell Mol Neurobiol. 2021;41:1271–84. 32529543 10.1007/s10571-020-00898-z PMC11448642

[14] Zhou L, Li J, Liao M, Zhang Q, Yang M. LncRNA MIR155HG induces M2 macrophage polarization and drug resistance of colorectal cancer cells by regulating ANXA2. Cancer Immunol, Immunother. 2022;71:1075–91. 34562123 10.1007/s00262-021-03055-7 PMC10991596

[15] Peng L, Chen Z, Chen Y, Wang X, Tang N. MIR155HG is a prognostic biomarker and associated with immune infiltration and immune checkpoint molecules expression in multiple cancers. Cancer Med. 2019;8:7161–73. 31568700 10.1002/cam4.2583 PMC6885872

[16] Qiu X, Yang S, Wang S, Wu J, Zheng B, Wang K, et al. M(6)A Demethylase ALKBH5 Regulates PD-L1 Expression and Tumor Immunoenvironment in Intrahepatic Cholangiocarcinoma. Cancer Res. 2021;81:4778–93. 34301762 10.1158/0008-5472.CAN-21-0468

[17] Clark K, Vendt B, Smith K, Freymann J, Kirby J, Koppel P, et al. The Cancer Imaging Archive (TCIA): maintaining and operating a public information repository. J Digit imaging. 2013;26:1045–57. 23884657 10.1007/s10278-013-9622-7 PMC3824915

[18] Groom JR, Luster AD. CXCR3 ligands: redundant, collaborative and antagonistic functions. Immunol cell Biol. 2011;89:207–15. 21221121 10.1038/icb.2010.158 PMC3863330

[19] Mabrouk N, Tran T, Sam I, Pourmir I, Gruel N, Granier C, et al. CXCR6 expressing T cells: Functions and role in the control of tumors. Front Immunol. 2022;13:1022136. 36311728 10.3389/fimmu.2022.1022136 PMC9597613

[20] Kong W, Yang H, He L, Zhao JJ, Coppola D, Dalton WS, et al. MicroRNA-155 is regulated by the transforming growth factor beta/Smad pathway and contributes to epithelial cell plasticity by targeting RhoA. Mol Cell Biol. 2008;28:6773–84. 18794355 10.1128/MCB.00941-08 PMC2573297

[21] Bridges MC, Daulagala AC, Kourtidis A. LNCcation: lncRNA localization and function. J Cell Biol. 2021;220:e202009045. 10.1083/jcb.202009045 PMC7816648 33464299

[22] Lyabin DN, Eliseeva IA, Ovchinnikov LP. YB-1 protein: functions and regulation. Wiley Interdiscip Rev RNA. 2014;5:95–110. 24217978 10.1002/wrna.1200

[23] Cui Q, Wang C, Liu S, Du R, Tian S, Chen R, et al. YBX1 knockdown induces renal cell carcinoma cell apoptosis via Kindlin-2. Cell Cycle. 2021;20:2413–27. 34709966 10.1080/15384101.2021.1985771 PMC8794528

[24] Su H, Fan G, Huang J, Qiu X. LncRNA HOXC-AS3 promotes non-small-cell lung cancer growth and metastasis through upregulation of YBX1. Cell Death Dis. 2022;13:307. 35387975 10.1038/s41419-022-04723-x PMC8986809

[25] Ruan H, Bao L, Tao Z, Chen K. Flightless I homolog reverses enzalutamide resistance through PD-L1-mediated immune evasion in prostate cancer. Cancer Immunol Res. 2021;9:838–52. 34011528 10.1158/2326-6066.CIR-20-0729

[26] Budimir N, Thomas GD, Dolina JS, Salek-Ardakani S. Reversing T-cell exhaustion in cancer: lessons learned from PD-1/PD-L1 immune checkpoint blockade. Cancer Immunol Res. 2022;10:146–53. 34937730 10.1158/2326-6066.CIR-21-0515

[27] Tao Z, Ruan H, Sun L, Kuang D, Song Y, Wang Q, et al. Targeting the YB-1/PD-L1 axis to enhance chemotherapy and antitumor immunity. Cancer Immunol Res. 2019;7:1135–47. 31113805 10.1158/2326-6066.CIR-18-0648

[28] Peng J, Zou D, Gong W, Kang S, Han L. Deep neural network classification based on somatic mutations potentially predicts clinical benefit of immune checkpoint blockade in lung adenocarcinoma. Oncoimmunology. 2020;9:1734156. 32158626 10.1080/2162402X.2020.1734156 PMC7051190

[29] Wu W, Yu T, Wu Y, Tian W, Zhang J, Wang Y. The miR155HG/miR-185/ANXA2 loop contributes to glioblastoma growth and progression. J Exp Clin Cancer Res. 2019;38:133. 30898167 10.1186/s13046-019-1132-0 PMC6427903

[30] Petroni G, Buqué A, Coussens LM, Galluzzi L. Targeting oncogene and non-oncogene addiction to inflame the tumour microenvironment. Nat Rev Drug Discov. 2022;21:440–62. 35292771 10.1038/s41573-022-00415-5

[31] Liu H, Kuang X, Zhang Y, Ye Y, Li J, Liang L, et al. ADORA1 inhibition promotes tumor immune evasion by regulating the ATF3-PD-L1 axis. Cancer Cell. 2020;37:324–39.e328. 32183950 10.1016/j.ccell.2020.02.006

[32] Ji P, Gong Y, Jin ML, Wu HL, Guo LW, Pei YC, et al. In vivo multidimensional CRISPR screens identify Lgals2 as an immunotherapy target in triple-negative breast cancer. Sci Adv. 2022;8:eabl8247. 35767614 10.1126/sciadv.abl8247 PMC9242595

[33] Wang J, Wang Q, Guan Y, Sun Y, Wang X, Lively K, et al. Breast cancer cell-derived microRNA-155 suppresses tumor progression via enhancing immune cell recruitment and antitumor function. J Clin Investig. 2022;132:e157248. 10.1172/JCI157248 PMC9525116 35925680

[34] Reina-Campos M, Scharping NE, Goldrath AW. CD8(+) T cell metabolism in infection and cancer. Nat Rev Immunol. 2021;21:718–38. 33981085 10.1038/s41577-021-00537-8 PMC8806153

[35] Leclerc M, Voilin E, Gros G, Corgnac S, de Montpréville V, Validire P, et al. Regulation of antitumour CD8 T-cell immunity and checkpoint blockade immunotherapy by Neuropilin-1. Nat Commun. 2019;10:3345. 31350404 10.1038/s41467-019-11280-z PMC6659631

[36] Paijens ST, Vledder A, de Bruyn M, Nijman HW. Tumor-infiltrating lymphocytes in the immunotherapy era. Cell Mol Immunol. 2021;18:842–59. 33139907 10.1038/s41423-020-00565-9 PMC8115290

[37] Francisco LM, Sage PT, Sharpe AH. The PD-1 pathway in tolerance and autoimmunity. Immunol Rev. 2010;236:219–42. 20636820 10.1111/j.1600-065X.2010.00923.x PMC2919275

[38] Li R, Huang X, Yang W, Wang J, Liang Y, Zhang T, et al. Tertiary lymphoid structures favor outcome in resected esophageal squamous cell carcinoma. J Pathol Clin Res. 2022;8:422–35. 35711130 10.1002/cjp2.281 PMC9353661

