# Supplementary material for: The recruitment of CD8+ T cells through YBX1 stabilization abrogates tumor intrinsic oncogenic role of MIR155HG in lung adenocarcinoma
Source: Cell Death Discov. 2024 Jul 23;10:334. doi: 10.1038/s41420-024-02102-3 (PMC11266398; doi:10.1038/s41420-024-02102-3)
Supplement: Supplementary file 1 — Supplementary Matrials [file 41420_2024_2102_MOESM1_ESM.docx]

**Supplementary Materials**

**The recruitment of CD8^+^ T cells through YBX1 stabilization abrogates** **tumor intrinsic oncogenic role of** **MIR155HG in lung adenocarcinoma**

**Rutao Li^1,2,3*^, Yijian Zhang^1,2,3*^, Anpeng Wang^4*^, Yipeng Feng^1,2,3^, Te zhang^1,2,3^, Hui Wang^1,2,3^, Yuzhong Chen^1,2,3^, Xinnian Yu^2,3,5^, Xuming Song^1,2,3^, HanLin Ding^1,2,3^, Lin Xu^1,2,3,6^, Gaochao Dong^1,2,3#^, Feng Jiang^1,2,3#^**

1. Department of Thoracic Surgery, Nanjing Medical University Affiliated Cancer Hospital & Jiangsu Cancer Hospital & Jiangsu Institute of Cancer Research, Nanjing, China.
2. Jiangsu Key Laboratory of Molecular and Translational Cancer Research, Cancer Institute of Jiangsu Province, Nanjing, China.
3. The Fourth Clinical College of Nanjing Medical University, Nanjing, China.
4. Department of Geriatric Oncology, The First Affiliated Hospital of Nanjing Medical University, Nanjing, China
5. Department of Oncology, Nanjing Medical University Affiliated Cancer Hospital & Jiangsu Cancer Hospital & Jiangsu Institute of Cancer Research, Nanjing, China.
6. Collaborative Innovation Center for Cancer Personalized Medicine, Nanjing Medical University, Nanjing, China.

*Rutao Li, Yijian Zhang and Anpeng Wang contributed equally to this word.

**^#^Corresponding authors:** Feng Jiang and Gaochao Dong

**Feng Jiang**

Department of Thoracic Surgery, Jiangsu Key Laboratory of Molecular and Translational Cancer Research, Nanjing Medical University Affiliated Cancer Hospital & Jiangsu Cancer Hospital & Jiangsu Institute of Cancer Research, Nanjing, China.

E-mail addresses: [fengjiang_nj@njmu.edu.cn](mailto:fengjiang_nj@njmu.edu.cn).

**Gaochao Dong**

Department of Thoracic Surgery, Jiangsu Key Laboratory of Molecular and Translational Cancer Research, Nanjing Medical University Affiliated Cancer Hospital & Jiangsu Cancer Hospital & Jiangsu Institute of Cancer Research, Nanjing, China.

E-mail addresses: [gaochao_dong@njmu.edu.cn](mailto:gaochao_dong@njmu.edu.cn).

**Competing Interests**

The authors have no relevant financial or non-financial interests to disclose.

**Contents**

Supplementary Figure S1: MIR155HG is upregulated in LUAD tissues and correlated with good prognosis.

Supplementary Figure S2: MIR155HG is associated with several immune cells and its function is independent of miR-155.

Supplementary Figure S3: MIR155HG is subcellular in the cytoplasm and interacts with YBX1.

Supplementary Figure S4: MIR155HG is associated with multiple checkpoints and promotes PD-L1 transcription through YBX1 in LUAD.

Supplementary Figure S5: MIR155HG correlates with immunophenotypic Score and improves anti-tumor effect of PD-L1 blockade in vivo.

Supplementary Table 1: Primer sets, Sequences of siRNAs and shRNA sets

Supplementary Table 2: MIR155HG-related gene signature with 100 genes correlated with MIR155HG

Supplementary Table 3: DEGs in MIR155G-overexpressed A549 cells compared with parental A549 cells by RNA-sequence.

**Figure S1: MIR155HG is upregulated in LUAD tissues and correlated with good prognosis.** A. Relative expression levels of MIR155HG in LUAD tissues and normal tissues in GSE40791. B. Kaplan–Meier survival curves of survival probability according to MIR155HG-related gene signature in several LUAD cohorts. C. qRT-PCR detection of MIR155HG expression in multiple LUAD cells and in normal lung bronchial epithelial cell. D. Validation of the knockdown and overexpression efficacy of MIR155HG in A549 and PC9 cell lines by qRT-PCR. G: Representative images MIR155HG on tumor formation in BALB/c nude mouse xenograft model. F. Effect of MIR155HG on tumor formation in PBMC-transferred NCG mouse xenograft model. Representative images of tumors from sh-MIR155HG and control groups /MIR155HG-overexpressed and control groups (n=5). Results are presented as mean ± SEM, n = 3. **p < 0.01, ***p < 0.001.

**Figure S2: MIR155HG is associated with several immune cells and its function is independent of miR-155.** A. Correlation between MIR155HG level and immune filtrates. Data were derived from TIMER 2.0 (<http://timer.comp-genomics.org/>). B. miR-155 inhibitor has no effect on increased recruitment of CD3+CD8+ T cells in MIR155HG-overexpressing A549 cells by flow cytometry analysis. Results are presented as mean ± SEM, n = 3. ns, not significant, ***p < 0.001.

**Figure S3: MIR155HG is subcellular in the cytoplasm and interacts with YBX1.** A. Nucleocytoplasmic separation assays of MIR155HG in A549 and PC9 cells. Cytosolic and nuclear markers include GAPDH and U6. B. FISH of MIR155HG in A549 and PC9 cells; scale bars, 20 μm. C. Summary of mass spectrometry results of MIR155HG pull-down. D. The potential YBX1-binding region of MIR155HG was predicted using catRAPI omics. E. Knockdown and overexpression of MIR155HG have no significant effect on the mRNA levels of YBX1 in A549 and PC9 cells.

F. Quantification of YBX1 protein levels with knockdown and overexpression of MIR155HG by Western Blot, G. Validation of the knockdown efficacy of YBX1 in A549 cell lines by qRT-PCR. Results are presented as mean ± SEM, n = 3. ns, not significant; *p < 0.05, **p < 0.01, ***p < 0.001.

**Figure S4: MIR155HG is associated with multiple checkpoints and promotes PD-L1 transcription through YBX1 in LUAD.** A. Summary of Spearman correlations between MIR155HG and immune checkpoints mRNA levels in TCGA dataset. B. Correlations of MIR155HG levels with PD-1, CTLA-4, LAG3 and TIM3 expression in PBMC. C. Correlations of MIR155HG levels with PD-L1 expression in LUAD tissues.

D. Quantification of PD-L1 protein levels with knockdown and overexpression of MIR155HG by Western Blot. E. PD-L1 reversed the decrease of TOX mRNA expression induced by MIR155HG. F. The human PD-L1 proximal promoter (-1~-1.5 kb) was divided into seven different segments. G. ChIP analysis was used to study the potential binding of YBX1 in A549 cells. YBX1 bound to the designated regions (P6, containing the Y-box sequence). ChIP assays were performed using a YBX1-specific antibody, R-IgG was the ChIP control. H. Three different luciferase vectors constructed according to PD-L1 promoter P6 region were co-transfected with si-YBX1 into A549 cells. The relative firefly/Renilla luciferase activities were analyzed in the cells 24 h after transfection. Results are presented as mean ± SEM, n = 3. ns, not significant; *p < 0.05, **p < 0.01, ***p < 0.001.

**Figure S5: MIR155HG correlates with immunophenotypic Score and improves anti-tumor effect of PD-L1 blockade in vivo.** A. Representative images of immunophenotypic Score in LUAD patients. B. Relative IPS in MIR155HGhi and MIR155HGlo LUAD patients. C. Tumor growth curves and tumor volumes of sh-NC/sh-MIR155HG-transfected A549 cells with PD-L1 mAb treatment in PBMC-transferred NCG mice. D. Effect of MIR155HG on tumor formation in PBMC-transferred NCG mouse xenograft model with PD-L1 mAb treatment. Representative images of tumors from sh-MIR155HG and control groups (n=5). E. Representative images of tumors from MIR155HG-overexpressed and control groups (n=5). Results are presented as mean ± SEM, n = 3. ns, not significant; *p < 0.05, **p < 0.01, ***p < 0.001.

**Supplementary Figure S1: MIR155HG is upregulated in LUAD tissues and correlated with good prognosis.**


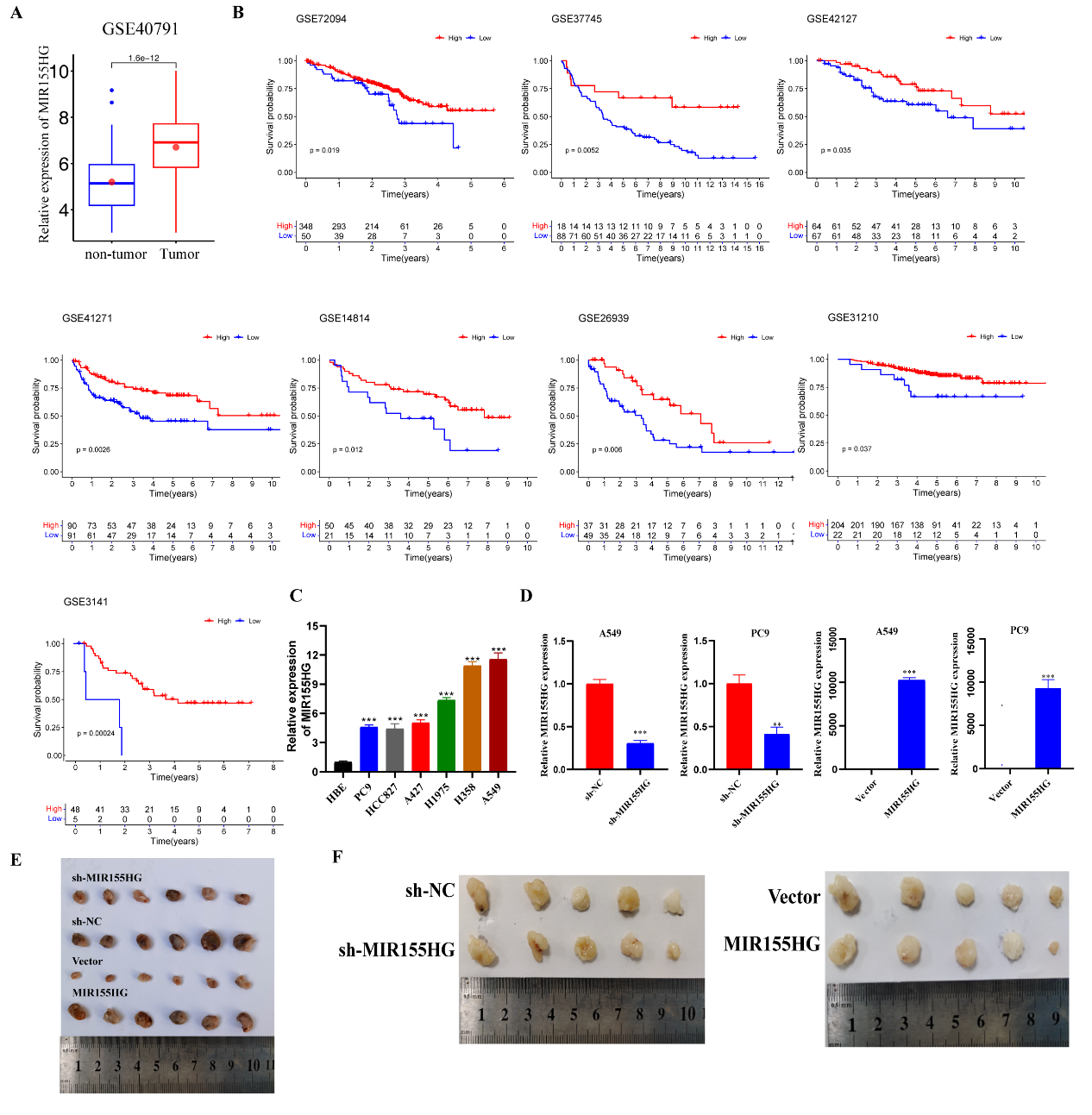


**Supplementary Figure S2: MIR155HG is associated with several immune cells and its function is independent of miR-155.**


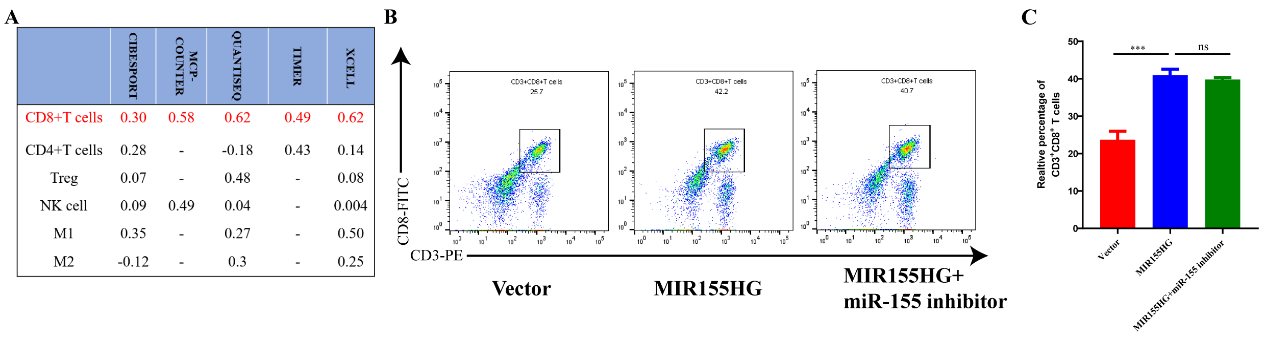


**Supplementary Figure S3: MIR155HG is subcellular in the cytoplasm and interacts with YBX1**


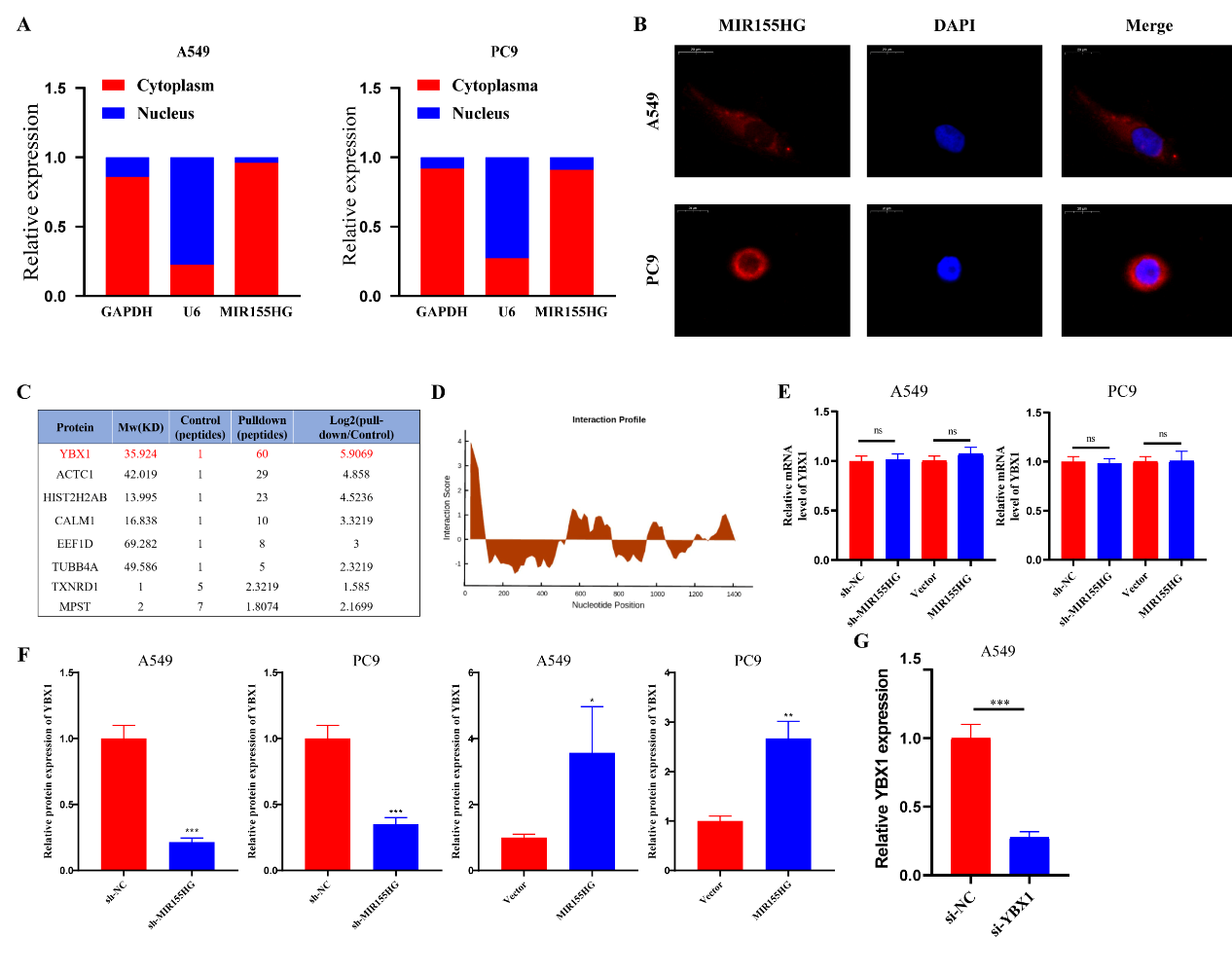


**Supplementary Figure S4: MIR155HG is associated with multiple checkpoints and promotes PD-L1 transcription through YBX1 in LUAD**


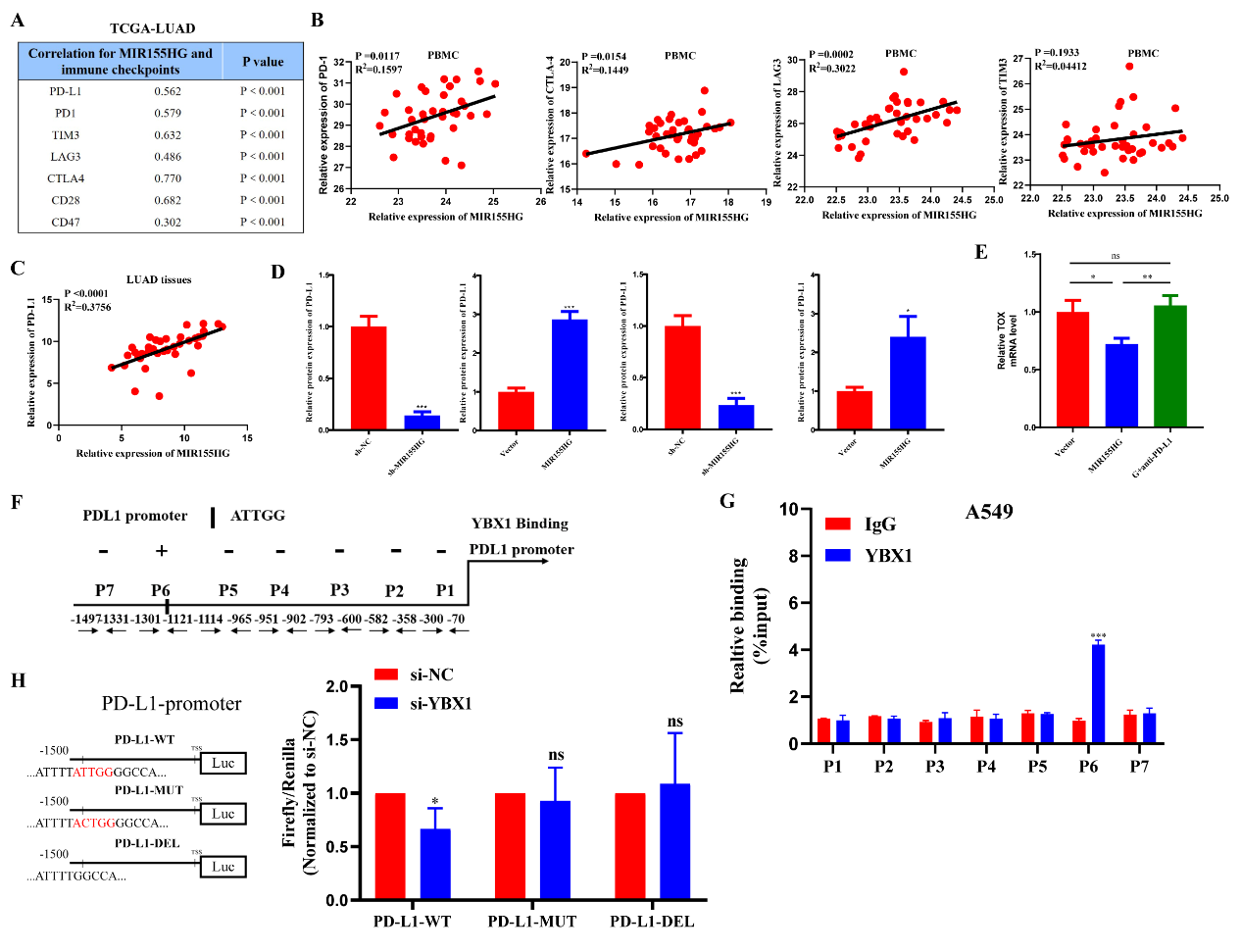


**Supplementary Figure S5: MIR155HG correlates with immunophenotypic Score and improves anti-tumor effect of PD-L1 blockade in vivo.**


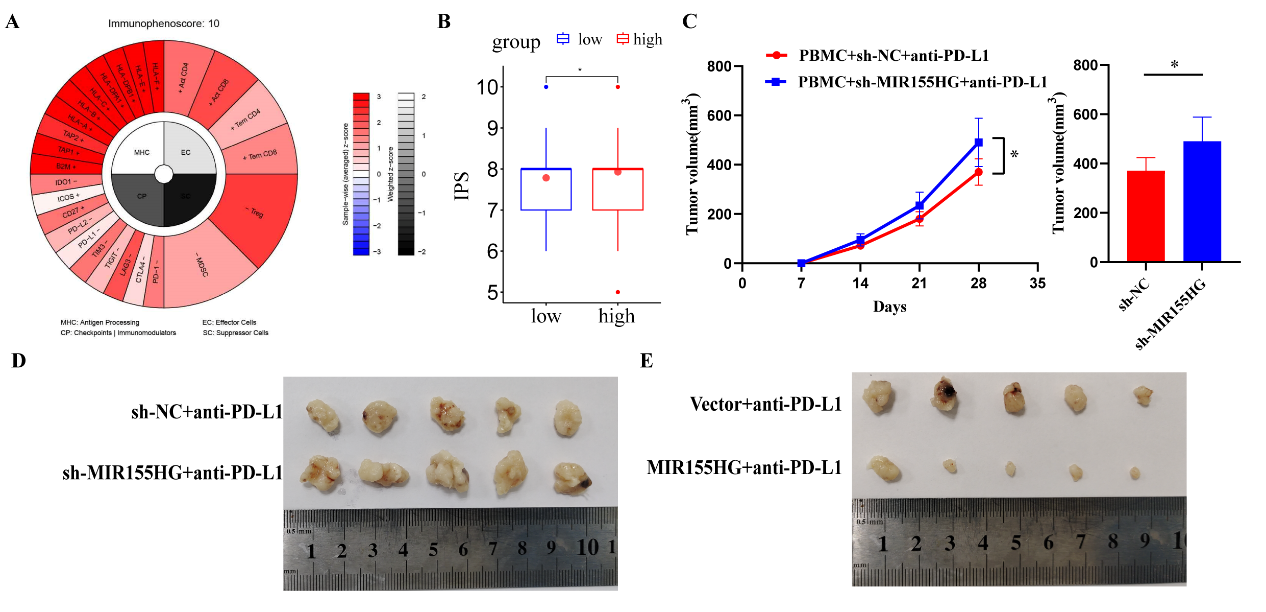


**Supplementary Table 1: Primer sets, Sequences of siRNAs and shRNA sets**

| Primer set | Primers | Sequence |  |
| --- | --- | --- | --- |
| MIR155HG | Forward | 5’-GAGTGCTGAAGGCTTGCTGT-3’ |  |
|  | Reverse | 5’-TTGAACATCCCAGTGACCAG-3’ |  |
|  | Reverse | 5’-ACCCGTGGTCACCATGGTA-3’ |  |
| U6 | Forward | 5’-CTCGCTTCGGCAGCACA-3’ |  |
|  | Reverse | 5’-AACGCTTCACGAATTTGCG-3’ |  |
| GAPDH | Forward | 5’-AGAAGGCTGGGGCTCATTTG-3’ |  |
|  | Reverse | 5’-AGGGGCCATCCACAGTCTTC-3’ |  |
| CCL5 | Forward | 5’-CCAGCAGTCGTCTTTGTCAC-3’ |  |
|  | Reverse | 5’-CTCTGGGTTGGCACACACTT-3’ |  |
| CXCL1 | Forward | 5’-TGCTGCCACTAATGCTGATGT -3’ |  |
|  | Reverse | 5’-CTCAGGAACCAATCTTTGCACT -3’ |  |
| CXCL2 | Forward | 5’-CCAACCACCAGGCTACAGG-3’ |  |
|  | Reverse | 5’-GCGTCACACTCAAGCTCTG-3’ |  |
| CXCL5 | Forward | 5’-AGCTGCGTTGCGTTTGTTTAC -3’ |  |
|  | Reverse | 5’-TGGGAACACTTGCAGATTAC-3’ |  |
| CXCL8 | Forward | 5’-TTTTGCCAAGGAGTGCTAAAGA -3’ |  |
|  | Reverse | 5’-AACCCTCTGCACCCAGTTTTC-3’ |  |
| CXCL12 | Forward | 5’-ATTCTCAACACTCCAAACTGTGC-3’ |  |
|  | Reverse | 5’-ACTTTAGCTTCGGGTCAATGC-3’ |  |
| CCL2 | Forward | 5’-CATCTCCTACACCCCACGAAG-3’ |  |
|  | Reverse | 5’-GGGTTGGCACAGAAACGTC-3’ |  |
| CCL17 | Forward | 5’-TACCATGAGGTCACTTCAGATGC-3’ |  |
|  | Reverse | 5’- GCACTCTCGGCCTACATTGG-3’ |  |
| CCL22 | Forward | 5’-ATCGCCTACAGACTGCACTC-3’ |  |
|  | Reverse | 5’-GACGGTAACGGACGTAATCAC-3’ |  |
| CCL28 | Forward | 5’-TGCACGGAGGTTTCACATCAT -3’ |  |
|  | Reverse | 5’-TTGGCAGCTTGCACTTTCATC -3’ |  |
| YBX1 | Forward | 5’-TAGACGCTATCCACGTCGTAG-3’ |  |
|  | Reverse | 5’-ATCCCTCGTTCTTTTCCCCAC-3’ |  |
| PDL1 | Forward | 5’-GCTGGATTACGTCTCCTCCA-3’ |  |
|  | Reverse | 5’-GTGGCATCCAAGATACAAACTCA-3’ |  |
| C1 | Forward | 5’-GTCCTTCCCCATGAGCATTA-3’ |  |
|  | Reverse | 5’-CCCCTCCAGAGTGGGTAAGT-3’ |  |
| C2 | Forward | 5’-TTCTTTCTCTGACGGACTTGC-3’ |  |
|  | Reverse | 5’-CCATGAGCATTAGGCACACA-3’ |  |
| C3 | Forward | 5’-TTCCCAAGCTTGTCCCTTTA-3’ |  |
|  | Reverse | 5’-CTCTTGCTAAGGGTTTTCCTGA-3’ |  |
| C4 | Forward | 5’-AGGAGAATGGCGTGAACCT-3’ |  |
|  | Reverse | 5’-AAAGCATGGATTTTGGCATT-3’ |  |
| C5 | Forward | 5’-GGGCAGTGTGTGGTCAGTAT-3’ |  |
|  | Reverse | 5’-TCCTGAGTATGGTATGCATGTTG-3’ |  |
| C6 | Forward | 5’-AGGAAGACAGACCTGTGAGCA-3’ |  |
|  | Reverse | 5’-TTTATTCTCCCTCAAGGGTTCC-3’ |  |
| C7 | Forward | 5’-ACAGCAAACCACCATGACAC-3’ |  |
|  | Reverse | 5’-ACATACCCTCTCCCCGTCTT-3’ |  |
| IFNG | Forward | 5’-TCGCTTCCCTGTTTTAGCTGC-3’ |  |
|  | Reverse | 5’-TCGGTAACTGACTTGAATGTCCA-3’ |  |
| GNLY | Forward | 5’-CAGGCTCCCTGCCCATAAAA-3’ |  |
|  | Reverse | 5’-GGACATGAGGTCATACGTGGAG-3’ |  |
| GZMB | Forward | 5’-CTCAAGGCCTGGGTTGCC-3’ |  |
|  | Reverse | 5’-GCACAACTCAATGGTACTGTCG-3’ |  |
| PRF1 | Forward | 5’-GGCTGGACGTGACTCCTAAG-3’ |  |
|  | Reverse | 5’-CTGGGTGGAGGCGTTGAAG-3’ |  |
| P1 | Forward | 5’ GTTGTAGACTTGCGTGGAAC-3’ |  |
|  | Reverse | 5’-CTAAAGTGGCTTCCAGTCCT-3’ |  |
| P2 | Forward | 5’-AGCACCTAAGACACTGAAGGA-3’ |  |
|  | Reverse | 5’-AAAATAGTCTTTCCCCCTGC-3’ |  |
| P3 | Forward | 5’-TTCATAATAGGGCGCGACTT-3’ |  |
|  | Reverse | 5’-CATCGAGCCCTACCCTTCAA-3’ |  |
| P4 | Forward | 5’-AGCAACAAACGGAAAGGAAG-3’ |  |
|  | Reverse | 5’-CCTCAAGCTCTTTTCTCTCGT-3’ |  |
| P5 | Forward | 5’-TAACTCCGACTGTGACGGAA-3’ |  |
|  | Reverse | 5’-AATACGTCATAAAACTTGACGTC-3’ |  |
| P6 | Forward | 5’- ACTGAAGGGTTGAAACCACT-3’ |  |
|  | Reverse | 5’- TCTCTTGAGGTACGAGGACG-3’ |  |
| P7 | Forward | 5’- AGTTCTGGGTCCCGTATTTG-3’ |  |
|  | Reverse | 5’-TGGACATTTGACATAACGGTGT-3 |  |

| Oligo Set | Sequences |
| --- | --- |
| pcDNA3.1(+)- | 5’-tactaatgactttttttttatacttcagATTTTCAGCTGAACTCTCAT-3’(sense) |
| Native Control | UUCUCCGAACGUGUCACGUTT（sense）  ACGUGACACGUUCGGAGAATT（antisense） |
| sh-MIR155HG | CUGGGAUGUUCAACCUUAATT |
| si-YBX1 | CCACGCAATTACCAGCAAA |
| si-PDL1 | GTGACCAGCACACTGAGAA |
| MIR155HG-CY3 | TGATA+TAAC+TGGAGGT+TAG+TAG+TCC |

**Supplementary Table 2：MIR155HG-related gene signature with 100 genes correlated with MIR155HG**

|  | pvalue | cor |  | pvalue | cor |
| --- | --- | --- | --- | --- | --- |
| ARHGAP9 | 1.99E-119 | 0.809218 | C5orf56 | 2.24E-80 | 0.713063 |
| TBC1D10C | 4.55E-119 | 0.808525 | GZMA | 2.37E-80 | 0.712989 |
| TRAF3IP3 | 7.85E-119 | 0.808065 | SLAMF6 | 3.64E-80 | 0.712407 |
| SH2D1A | 3.88E-116 | 0.802743 | GPR174 | 4.01E-80 | 0.712276 |
| PTPN7 | 8.08E-111 | 0.79174 | GFI1 | 8.96E-80 | 0.711181 |
| ACAP1 | 1.95E-110 | 0.790922 | CD53 | 1.04E-79 | 0.71098 |
| PSTPIP1 | 3.77E-109 | 0.788144 | TTC24 | 1.62E-79 | 0.71037 |
| CD247 | 4.60E-109 | 0.787956 | SLFN12L | 1.65E-79 | 0.710344 |
| CD2 | 1.61E-107 | 0.78456 | BTK | 2.56E-79 | 0.709741 |
| CD3D | 2.74E-104 | 0.777255 | NLRC3 | 1.05E-78 | 0.707801 |
| SIT1 | 5.76E-104 | 0.776509 | CCR5 | 3.77E-78 | 0.706017 |
| ZAP70 | 2.83E-101 | 0.770176 | SNX20 | 1.06E-77 | 0.704566 |
| ICOS | 3.13E-101 | 0.770071 | SLA2 | 1.77E-77 | 0.703851 |
| PYHIN1 | 5.50E-100 | 0.767072 | CD8A | 1.18E-75 | 0.697862 |
| SIRPG | 8.95E-100 | 0.766557 | MCOLN2 | 1.43E-75 | 0.697587 |
| CLEC2D | 9.05E-100 | 0.766546 | CORO1A | 2.35E-75 | 0.696862 |
| SLAMF1 | 1.20E-99 | 0.766246 | PTPRC | 5.11E-75 | 0.695735 |
| IL18RAP | 1.11E-96 | 0.758882 | IL10RA | 7.01E-75 | 0.695275 |
| GPR18 | 1.77E-96 | 0.758368 | CCL4 | 1.60E-74 | 0.694066 |
| CTLA4 | 3.09E-95 | 0.755192 | GIMAP5 | 2.67E-74 | 0.693315 |
| TRAT1 | 3.26E-95 | 0.755132 | CXorf65 | 2.72E-74 | 0.693286 |
| CD48 | 4.34E-94 | 0.752217 | LCP2 | 1.41E-73 | 0.690854 |
| IL12RB1 | 3.72E-92 | 0.747104 | IL16 | 2.66E-73 | 0.68991 |
| TIGIT | 4.06E-91 | 0.744308 | CD6 | 6.70E-73 | 0.688526 |
| SASH3 | 1.22E-90 | 0.743009 | IL21R | 1.04E-72 | 0.68786 |
| CRTAM | 1.47E-89 | 0.740041 | EVI2B | 1.10E-72 | 0.687782 |
| CD3E | 2.34E-89 | 0.739479 | BIN2 | 1.12E-72 | 0.687754 |
| MAP4K1 | 3.09E-89 | 0.739144 | ITGAL | 1.84E-72 | 0.687009 |
| LTA | 6.87E-89 | 0.738179 | GBP5 | 2.46E-72 | 0.686566 |
| BTLA | 1.31E-88 | 0.737392 | NCF1 | 1.00E-71 | 0.684435 |
| CXCR6 | 7.82E-88 | 0.735213 | CCL5 | 1.01E-71 | 0.684418 |
| LCK | 1.10E-87 | 0.734789 | PDCD1 | 1.84E-71 | 0.683502 |
| RASAL3 | 1.59E-87 | 0.73434 | TAGAP | 2.74E-71 | 0.682894 |
| UBASH3A | 2.81E-87 | 0.733638 | FASLG | 5.53E-71 | 0.681814 |
| GMFG | 3.22E-87 | 0.733466 | IRF8 | 1.07E-70 | 0.680794 |
| TFEC | 4.85E-87 | 0.732959 | FGD2 | 1.21E-70 | 0.680605 |
| GZMK | 7.71E-86 | 0.7295 | WAS | 8.30E-70 | 0.677604 |
| CD3G | 9.35E-86 | 0.729258 | NKG7 | 6.06E-69 | 0.674466 |
| SAMD3 | 7.73E-85 | 0.726576 | KLRB1 | 1.55E-68 | 0.672975 |
| CYTH4 | 1.32E-84 | 0.725889 | ZBTB32 | 2.48E-68 | 0.672221 |
| ITK | 1.74E-84 | 0.72554 | SAMSN1 | 3.60E-68 | 0.671624 |
| CD72 | 2.26E-84 | 0.7252 | GIMAP4 | 5.82E-68 | 0.670852 |
| THEMIS | 6.63E-84 | 0.723816 | TNFSF13B | 6.30E-68 | 0.670723 |
| CLECL1 | 9.25E-84 | 0.723385 | GIMAP7 | 1.00E-67 | 0.669975 |
| GPR65 | 1.09E-83 | 0.723176 | PLEK | 1.15E-67 | 0.66975 |
| ARHGAP15 | 7.01E-83 | 0.720747 | ABCD2 | 1.31E-67 | 0.66954 |
| APOBEC3G | 7.52E-83 | 0.720655 | ZBP1 | 1.37E-67 | 0.669474 |
| CD226 | 1.32E-82 | 0.719916 | LINC00158 | 1.54E-67 | 0.669283 |
| LPXN | 3.25E-82 | 0.718731 | HCST | 1.63E-67 | 0.669191 |
| NCR3 | 1.71E-80 | 0.713434 | P2RY10 | 2.00E-67 | 0.668857 |

**Supplementary Table 3: DEGs in MIR155G-overexpressed A549 cells compared with parental A549 cells by RNA-sequence.**

|  | baseMean(A549-Vector) | baseMean(A549-MIR155HG) | log2FoldChange | pvalue |
| --- | --- | --- | --- | --- |
| MIR155HG | 17.05057 | 63771.65 | 11.86106 | 0 |
| TNFSF12-TNFSF13 | 0 | 57.19022 | 8.26172 | 0.034523 |
| IFNL2 | 16.61612 | 121.0599 | 2.866148 | 4.14E-15 |
| XAF1 | 28.99117 | 196.7413 | 2.763575 | 5.56E-15 |
| GBP4 | 7.451893 | 44.75412 | 2.591805 | 3.53E-07 |
| SENP3-EIF4A1 | 1017.479 | 5881.859 | 2.531354 | 0.038303 |
| RSAD2 | 100.8743 | 569.2855 | 2.500051 | 1.43E-30 |
| IFIT2 | 595.6678 | 3085.904 | 2.374112 | 1.17E-48 |
| IFIT3 | 633.0226 | 2945.892 | 2.218551 | 3.18E-36 |
| OAS2 | 142.4498 | 659.5415 | 2.213648 | 2.06E-26 |
| IFNL1 | 29.56248 | 135.9569 | 2.206316 | 2.25E-14 |
| CMPK2 | 208.2882 | 932.6336 | 2.160943 | 1.05E-46 |
| TMEM88 | 10.5659 | 47.71617 | 2.16004 | 2.16E-05 |
| PCBP2-OT1 | 13.53197 | 59.94187 | 2.149827 | 6.00E-08 |
| H4C9 | 7.768262 | 34.00589 | 2.129211 | 0.00014 |
| H4C5 | 9.639775 | 41.28989 | 2.089356 | 1.46E-05 |
| IFI27 | 83.15792 | 351.6469 | 2.082103 | 2.76E-22 |
| ACAP1 | 246.7691 | 1026.92 | 2.057694 | 5.25E-19 |
| MX1 | 780.2334 | 3167.443 | 2.022046 | 2.70E-52 |
| LOC101930665 | 15.78393 | 62.80567 | 1.993852 | 7.97E-07 |
| IFNL3 | 28.63231 | 112.8592 | 1.975699 | 2.25E-09 |
| OASL | 507.1735 | 1940.837 | 1.935073 | 9.86E-35 |
| LOC105370877 | 18.17614 | 69.28124 | 1.929407 | 7.29E-08 |
| ISG15 | 1223.739 | 4632.797 | 1.920403 | 2.78E-43 |
| EGR1 | 810.7513 | 3051.29 | 1.912087 | 3.61E-71 |
| TRIM22 | 48.55874 | 181.9958 | 1.909721 | 6.74E-15 |
| MX2 | 29.12199 | 106.9211 | 1.885519 | 8.75E-09 |
| INSM2 | 22.37866 | 82.25657 | 1.883383 | 1.63E-06 |
| TNFSF10 | 17.97016 | 64.26518 | 1.852408 | 1.75E-05 |
| PARP10 | 161.7798 | 581.0239 | 1.842756 | 1.34E-20 |
| BATF2 | 291.8683 | 1046.785 | 1.842135 | 1.76E-38 |
| EGR2 | 15.73076 | 54.30744 | 1.785022 | 1.30E-05 |
| BCYRN1 | 342.9067 | 1181.255 | 1.784402 | 0.011638 |
| DLL1 | 18.58306 | 62.71097 | 1.752571 | 0.000282 |
| RPS20P27 | 27.15936 | 91.14625 | 1.749704 | 1.08E-07 |
| ACHE | 73.82641 | 247.8358 | 1.746464 | 3.81E-17 |
| IFI44L | 14.29403 | 47.61219 | 1.744155 | 5.05E-05 |
| UBA7 | 16.546 | 55.49269 | 1.741807 | 2.88E-05 |
| PATL2 | 14.88717 | 49.114 | 1.731949 | 0.000445 |
| TMEM140 | 34.02614 | 113.478 | 1.73062 | 1.69E-07 |
| CD300LB | 24.38554 | 80.22231 | 1.724255 | 5.69E-07 |
| HERC5 | 547.9075 | 1799.459 | 1.716558 | 8.54E-28 |
| SPEF1 | 26.26598 | 85.84202 | 1.713102 | 2.57E-06 |
| GAD1 | 12.00783 | 39.29091 | 1.704663 | 0.000178 |
| LOC100507336 | 12.15527 | 39.01528 | 1.686954 | 0.000419 |
| DLX2 | 156.1526 | 496.8376 | 1.669602 | 7.66E-26 |
| BTG3-AS1 | 18.99022 | 60.15769 | 1.66898 | 2.04E-05 |
| LOC107986982 | 16.781 | 53.72416 | 1.668341 | 0.000486 |
| IFIT1 | 1793.4 | 5648.777 | 1.65555 | 4.49E-33 |
| NPM1P26 | 22.0623 | 68.38534 | 1.638112 | 1.85E-05 |
| THEMIS2 | 47.7225 | 148.8622 | 1.63663 | 2.92E-09 |
| LOC107985289 | 21.64761 | 67.44294 | 1.631826 | 9.33E-06 |
| IL6 | 24.49072 | 73.78867 | 1.599536 | 1.19E-05 |
| BST2 | 30.47686 | 91.3514 | 1.591495 | 1.17E-06 |
| CHMP4BP1 | 16.87932 | 50.0949 | 1.566113 | 0.000429 |
| PIGHP1 | 15.12738 | 44.11464 | 1.558752 | 0.001355 |
| IFI6 | 1177.69 | 3455.376 | 1.552715 | 8.36E-50 |
| FOSB | 216.8887 | 632.0965 | 1.54182 | 5.06E-22 |
| IFIH1 | 411.9698 | 1195.749 | 1.537791 | 1.47E-19 |
| CD274 | 19.53705 | 56.40544 | 1.53219 | 7.27E-05 |
| UBE2V1P1 | 12.95926 | 37.47228 | 1.526761 | 0.003213 |
| IFI44 | 68.26793 | 195.4603 | 1.520922 | 1.60E-06 |
| CCL5 | 150.9431 | 431.8314 | 1.513651 | 9.52E-14 |
| ATOH8 | 242.6596 | 71.90947 | -1.75494 | 1.33E-14 |
| MAN1C1 | 40.1589 | 11.53119 | -1.78001 | 0.001834 |
| NDUFC2-KCTD14 | 41.58188 | 0.721093 | -5.88162 | 0.041892 |
| BNC2 | 218567.4 | 172.3453 | -10.3114 | 0 |
