# Supplementary figures and images for: The recruitment of CD8+ T cells through YBX1 stabilization abrogates tumor intrinsic oncogenic role of MIR155HG in lung adenocarcinoma
Source: Cell Death Discov. 2024 Jul 23;10:334. doi: 10.1038/s41420-024-02102-3 (PMC11266398; doi:10.1038/s41420-024-02102-3)

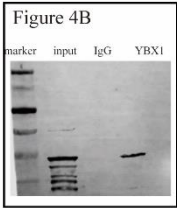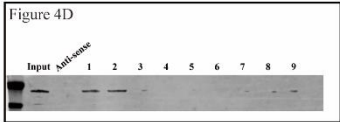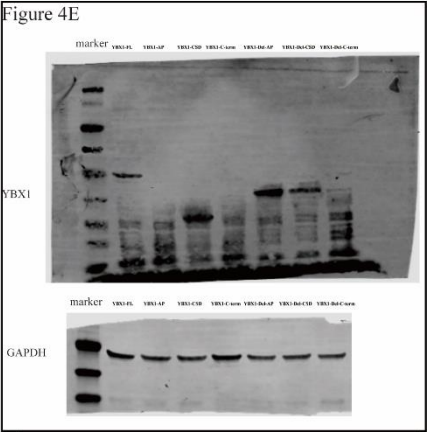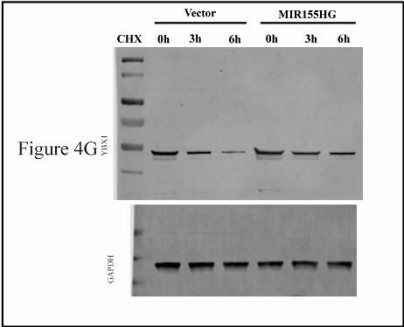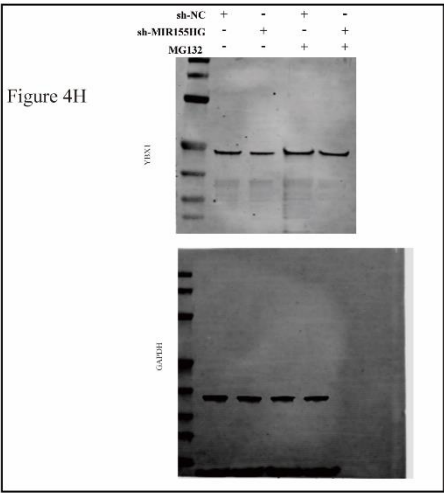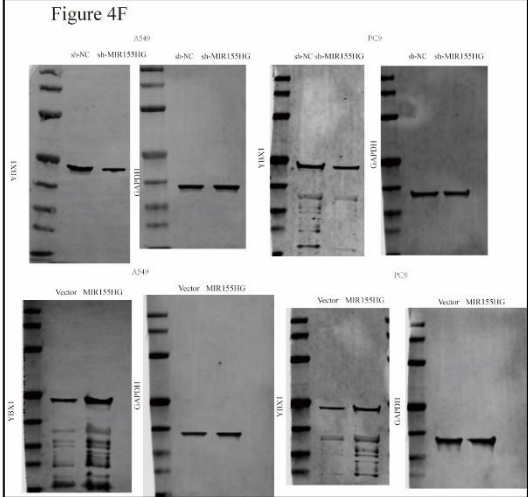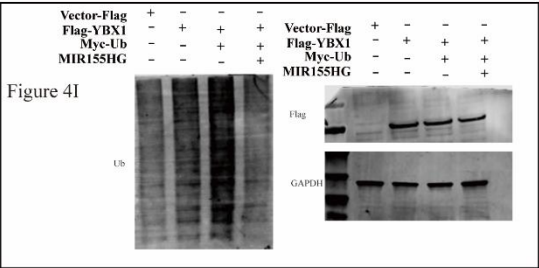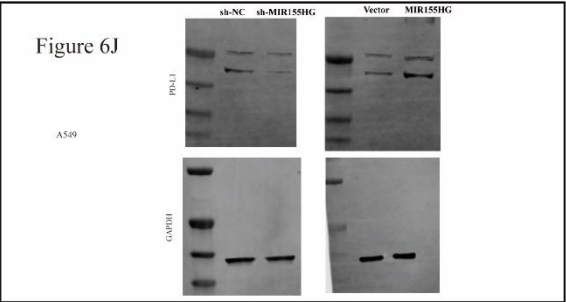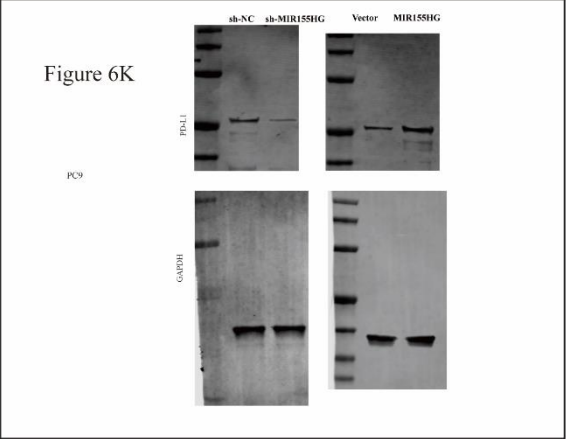

Supplement: Supplementary file 2 — original western blot [file 41420_2024_2102_MOESM2_ESM.pdf]
